# Estimating the number of hospital beds for the care of sick and small newborns: an evidence-based systematic approach

**DOI:** 10.7189/jogh.15.04312

**Published:** 2025-10-14

**Authors:** Bireshwar Sinha, Mohan Kumar, Deena Thomas, Natalie Strobel, Gagan Gupta, Karen Edmond, M Jeeva Sankar

**Affiliations:** 1 Society for Applied Studies, New Delhi, India; 2 Department of Community Medicine, KMCH Institute of Health Sciences and Research, Coimbatore, India; 3 Department of Pediatrics, Government Medical College, Kottayam, India; 4 Kurongkurl Katitjin | Centre for Indigenous Australian Education and Research, Edith Cowan University, Perth, Australia; 5 Health Programme, UNICEF, New York, NY, USA; 6 World Health Organization, Newborn and Child Health and Development Unit, Geneva, Switzerland; 7 Department of Pediatrics, All India Institute of Medical Sciences, New Delhi, India

## Abstract

**Background:**

Current recommendations for neonatal bed requirements are largely assumption-based rather than data-driven. We aimed to estimate the number of beds per 1000 live births needed for the care of small and sick newborns.

**Methods:**

We first extracted data from studies published between 2018 and May 2023. Then, due to considerable heterogeneity in the data, we performed a meta-analysis using a random effects model to estimate the number of neonatal admissions and the length of stay. We divided the total patient days (admission rate multiplied by the length of stay) by 365 to estimate the annual number of beds per 1000 live births.

**Results:**

We include 54 included studies, of which 46 provided data on the incidence of neonatal admissions and 20 on length of stay. The pooled analysis indicated that the number of neonates requiring admission ranged from 126 to 143 per 1000 live births. Admission rates were higher in the African region (160.5; 95% confidence interval (CI) = 122.2–198.7), in low-income countries (175.3; 95% CI = 102.8 to 247.8), in tertiary care settings (147.5; 95% CI = 115.9–179.1), and in settings with a high neonatal mortality rate (149.4; 95% CI = 90.5–218.2). The pooled length of stay was estimated to be 6.4 days (95% CI = 5.7–7.1). The overall estimated number of beds needed for the care of small and sick newborns was 2.4 (95% CI = 2.0–2.8) per 1000 live births, with regional variations.

**Conclusions:**

This method estimates the required neonatal care beds using admission rates and hospital stay data, aiding healthcare planning. Refinements and local adaptations are needed for effective policy decisions.

**Registration:**

PROSPERO: CRD42023417847

Small and/or sick newborns (SSNs) are infants who are either born preterm (before 37 weeks), have low birth weight (less than 2.5 kg), or become ill due to medical or surgical conditions within the first 28 days of life [1–3]. Target 4 of the global Every Newborn Action Plan aims to establish at least one level 2 small and sick newborn (SSN) care unit in every district (or equivalent) across all countries [3]. Considerable variation, however, exists in the population size of districts both within and between countries. For instance, a typical district (*woreda*) in Ethiopia has a population of about 200 000, while one in India has around 1.5 million residents. Birth rates also vary widely across and within countries, influencing the absolute number of births per district and, in turn, the demand for SSN care services. Estimating the number of beds for SSN care per 1000 live births offers a standardised and comparable metric to assess the expected burden on facility-based neonatal care systems.

Substantial progress has been made in defining standards for the availability of emergency obstetric care (EmOC), which encompasses both basic and comprehensive EmOC services. Comprehensive EmOC facilities, equipped to provide caesarean sections and blood transfusions, should ideally be available at district hospitals and level 2 health facilities. Current global recommendations call for a minimum of five EmOC facilities, *i.e.* four basic EmOC and one comprehensive EmOC facility for every 500 000 population, with the latter ideally located within a two-hour travel radius from a family’s home. These guidelines are derived from data collected in 2009 and are currently being reviewed in a process known as ‘EmOC re-visioning’ [4,5]. Notably, there remains a major gap in the development of equivalent benchmarks for facility-based newborn care, particularly in district hospitals in low- and middle-income countries [6]. The ongoing EmOC re-visioning process aims to address this by incorporating a stronger focus on newborn health, aligning with evolving global recommendations.

An assessment of special newborn care units (SNCUs) providing level 2 care in district hospitals across India in 2011 found significant operational challenges, most notably a widespread shortage of neonatal beds [6]. With rising admission rates, multiple neonates were accommodated in single beds, causing units to operate beyond advisable capacity. This strain was driven by increasing demand for facility-based neonatal care and the growing recognition of its role in improving newborn survival and outcomes. Previous estimates of the beds required for neonatal care varied from 0.7 to 6.5 beds per 1000 live births, yet they were based on assumptions that may no longer be valid [7]. Given these shortcomings, there is a pressing need to generate evidence-based estimates using real-world service data to inform neonatal care planning and adequate infrastructure to meet demand.

Our primary objective was to systematically estimate the total number of beds required for the care of SSNs per 1000 live births using available global data as of May 2023 on the incidence of neonatal admissions (which includes total admissions to the SNCU or neonatal intensive care units (NICUs)) and the length of hospital stay. We also sought to estimate the number of beds needed to care for SSNs per 1000 live births across six different WHO regions.

## METHODS

We employed a two-step approach to estimate the number of beds needed for the care of SSNs, defined as infants who are either born preterm (before 37 weeks), have low birth weight (less than 2.5 kg), or become ill due to medical or surgical conditions (respiratory distress, birth asphyxia, presumed or confirmed sepsis, severe jaundice requiring phototherapy or exchange transfusion, hypoglycaemia, seizures, major congenital anomaly, surgical emergency, or need for cardiorespiratory monitoring) within the first 28 days of life.

We first synthesised data on neonatal admission incidence rates per 1000 live births and the average length of hospital stay through a systematic review, and subsequently conducted a meta-analysis. Next, we calculated the total number of ‘patient days’ using the formula: patient days = (number of admissions per 1000 live births) × length of stay. To estimate the number of beds required per 1000 live births annually, we divided the ‘patient days’ by 365.

### Search strategy and selection criteria

To determine the total number of neonatal admissions in the SNCU or NICU, we first conducted an overview of systematic reviews and searched for available data sets at the regional, national, and sub-national levels from relevant governments or international organisations, such as the United Nations Children’s Fund. Due to the lack of existing systematic reviews, we conducted a new systematic review and meta-analysis to estimate the number of neonatal admissions to SNCUs or NICUs across various settings and the length of hospital stay. We searched Medline (*via* Ovid), Embase, and Cochrane CENTRAL for the period from 2018 to 3 May 2023 using a strategy that combined key terms and medical subject headings to ensure the inclusion of all studies involving newborns or neonates and any form of hospitalisation (Table S1 in the **Online Supplementary Document**). We did not apply any language restrictions. The titles, abstracts, and (if they were retained afterwards) full texts of the retrieved studies were screened by two people (NS, GG), with disagreements resolved by a third author. We excluded studies that reported neonatal admission rates without providing corresponding data on the number of live births in the population or the defined catchment area of the hospital, as this prevented calculation of standardised admission rates. 

### Data extraction

Two authors (MK, DT) independently extracted the following characteristics of the included studies: design (descriptive or experimental; cross-sectional or cohort), settings (hospital or community-based), study period, country, World Health Organization (WHO) region, and income status. For hospital-based studies, we also extracted the data on the level of healthcare facility (secondary or tertiary) and the type of neonatal unit (NICU, SNCU, or levels 1–4). We further recorded information on inborn SSNs neonatal admissions per 1000 live births. A correction factor was applied to adjust the estimates for studies providing hospital admission rates per 1000 deliveries. In these instances, we documented both the number of admissions and deliveries. We then estimated live births by adjusting for the stillbirth rate specific to that country and study period based on the WHO Global Health Observatory data repository [8], calculating the number of live births for these estimates per the formula (number of deliveries − (number of deliveries × stillbirth rate)). Any disagreements in the extraction were resolved through discussions with senior authors (BS or MJS).

We calculated the cumulative incidence of neonatal admissions to the SNCU or NICU by using the number of infants admitted before 28 days as the numerator and the total number of live births as the denominator, expressing the result per 1000 live births.

To obtain the pooled estimate for the length of stay (in days), we utilised the median duration of hospitalisation reported in the included studies, considering the typically positively skewed distribution of the variable. For studies that reported the length of stay as mean and standard deviation, we approximated the mean to the median and converted the standard deviation to standard error using the formula SD = SE × √N [9]. For studies that reported the median and interquartile range, we used the specified median and converted the interquartile range to standard error using the formula SE = (upper limit − lower limit)/3.92 [9]. For studies that reported length of stay categorised into various bands (*e.g.*<2, 2–5, 5 − 7, *etc*.), the categories were tabulated along with their proportions, and cumulative proportions were calculated. We averaged and approximated the limits of the categories containing the 50th percentile to the median, and the limits of the categories containing the 25th and 75th percentiles to the first and third quartiles, respectively. Using this interquartile range, we calculated the standard error as described above [10].

### Data synthesis

We performed the meta-analysis on neonatal admissions and length of stay using Stata, version 15.1 (StataCorp, College Station, Texas, USA). We intended to apply fixed-effect meta-analysis (inverse variance method) to combine data when it was reasonable to consider the studies homogeneous, but due to considerable heterogeneity in the estimates from various studies, we utilised a random effects model. We evaluated statistical heterogeneity among studies based on an *I*^2^ value >50% [9] and carried out subgroup analyses to explain it accordingly.

### Risk of bias assessment

Two review authors (MK, DT) independently evaluated the risk of bias of the included studies using the Joanna Briggs Institute critical appraisal checklist for studies reporting prevalence data [11]. A third review author (BS) was consulted in cases of discrepancies. Each item on the checklist received a score of either 1 (for ‘Yes’) or 0 (for ‘no’ or ‘unclear’), resulting in a maximum possible score of 9. Studies with scores ranging from 8 to 9 were classified as low risk, while those with scores below 8 were classified as high risk of bias (Table S4 in the **Online Supplementary Document**).

We also conducted a quality assessment using the Grading of Recommendations Assessment, Development, and Evaluation (GRADE) approach to evaluate the certainty of the pooled estimates of the number of neonatal admissions and the length of hospital stay [12].

### Subgroup analyses and assessment of publication bias

We conducted prespecified subgroup analysis according to the WHO regions (African Region, Region of the Americas, South-East Asian Region, European Region, Eastern Mediterranean Region, and Western Pacific Region); income status of countries (low-income, low- and middle-income, upper-middle-income, and high-income ); study setting (hospital or population-based); level of health facility (secondary or tertiary); risk of bias (low or high), and neonatal mortality rate (NMR) (≤12 or >12 per 1000 live births, defined according to Sustainable Development Goals target 3.2[13] per World Bank Data (2021)). We conducted meta-regression analyses to explore sources of heterogeneity, incorporating covariates such as WHO region, country income classification, study setting, level of health facility, and NMR. We used funnel plots and Egger’s test to determine the presence or absence of publication bias [14].

## RESULTS

**Figure 1:**
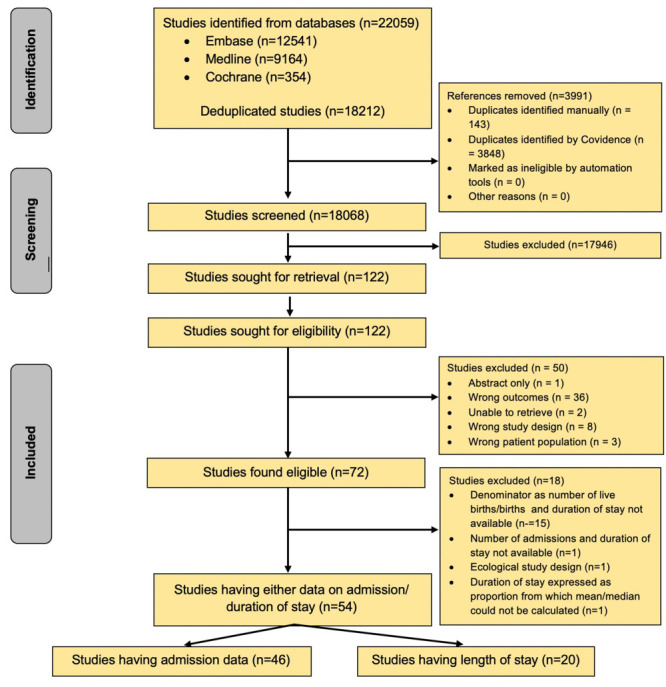
Adapted PRISMA flowchart for primary studies on hospital admissions.

The search yielded 18 212 unique records, of which 72 studies were eligible based on the screening process (**Figure 1**). Of these, 54 studies were included in the analyses that contained data on neonatal admissions or length of stay; 46 provided data on neonatal admissions [15–60] and 20 gave information on the length of hospital stay [16,17,19,21,26,28,32,35,41,47,53,55,61–68]. The studies represented all six WHO regions, with the majority from the African region and the Region of the Americas (**Table 1**). Three-fourths (73.9%) were hospital-based (11.8% secondary level and 88.2% tertiary level). Two-thirds (63.0%) were from low- or middle-income countries. More than one-third (34.8%) were from countries with a high NMR (>12 per 1000 live births). Most studies had a low risk of bias (82.6%).

**Table 1:** Characteristics of included studies (n = 46)

	n (%)
**Study setting**	
Hospital-based	34 (73.9)
Population-based	12 (26.1)
**Country’s income status**	
Low-income	6 (13.0)
Low- and middle-income	13 (28.3)
Upper middle-income	10 (21.7)
High-income	17 (37.0)
**Neonatal mortality rate in 2021**	
≤12	30 (65.2)
>12	16 (34.8)
**Level of health facility (n = 34)**	
Secondary	4 (11.8)
Tertiary	30 (88.2)
**World Health Organization region**	
Africa	12 (26.1)
South-East Asia	6 (13.0)
Western Pacific	3 (6.5)
Eastern Mediterranean	4 (8.7)
Europe	5 (10.9)
Region of the Americas	16 (34.8)

### Neonatal admissions

**Figure 2:**
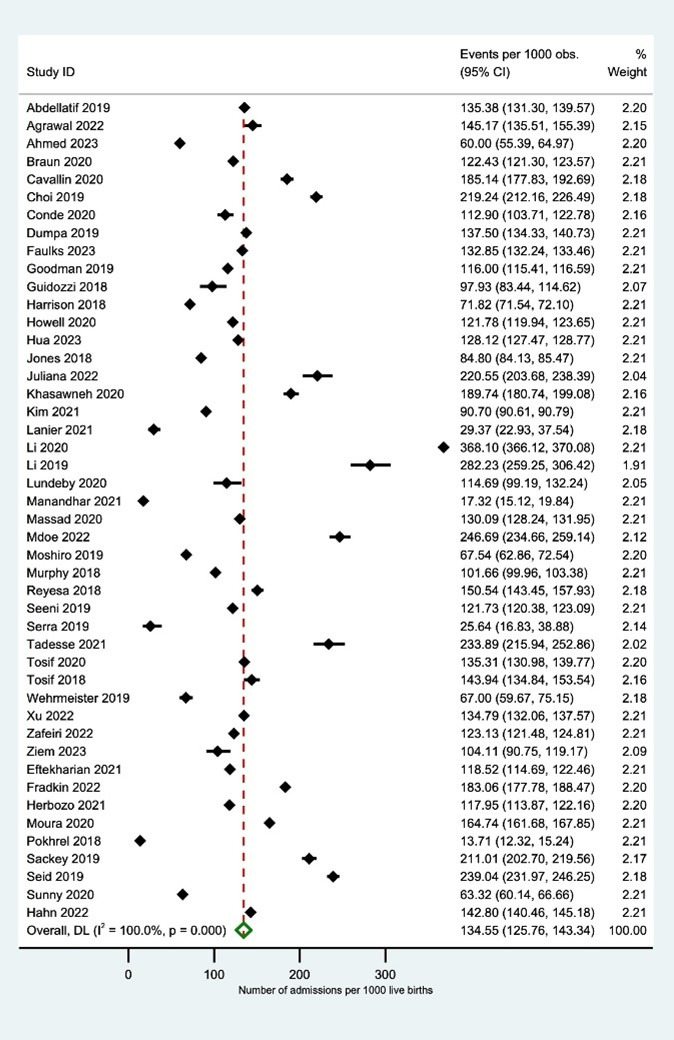
Overall number of neonatal admissions per 1000 live births.

Pooled analysis utilising the random effects model showed that the number of neonatal admissions was 134.6 (95% confidence interval (CI) = 125.8–143.3, *I*^2^ = 100%, 46 studies, n = 47 137 716 births, very low certainty of evidence) per 1000 live births (**Figure 2**, **Table 2**; Table S12 in the **Online Supplementary Document**).

**Table 2:** Number of beds for care of SSNs per 1000 live births

	Effect size (95% confidence interval)
Admission per 1000 live births	134.6 (125.8–143.3)
Length of stay in days	6.4 (5.7–7.1)
Beds per 1000 live births	2.4 (2.0–2.8)

Prespecified subgroup analyses revealed notable variation in neonatal admission rates per 1000 live births across regions, income groups, study settings, and health system characteristics (Figures S1–S5 and Tables S5–9 in the **Online Supplementary Document**). Admission rates were highest in the African Region (160.5; 95% CI = 122.2–198.7; *I*^2^ = 99.8%) and lowest in the European Region (96.5; 95% CI = 71.8–121.3, *I*^2^ = 100%). Low-income countries had a significantly higher admission rate (175.3; 95% CI = 102.8–247.8, *I*^2^ = 99.8%) than high-income countries (111.3; 95% CI = 101.9–120.8, *I*^2^ = 100%). Admissions were also more frequent in hospital-based studies (140.9; 95% CI = 111.8–170.1, *I*^2^ = 100%) than in population-based ones (117.7; 95% CI = 106.7–128.7, *I*^2^ = 100%). Within hospital-based studies, tertiary care facilities reported higher admission rates (147.5; 95% CI = 115.9–179.1, *I*^2^ = 100%) compared to secondary-level facilities (92.1; 95% CI = 42.6–141.6, *I*^2^ = 100%). Finally, settings with high NMRs had higher admission rates (149.4; 95% CI = 90.5–218.2, *I*^2^ = 100%) compared to those with lower NMRs (126.3; 95% CI = 118.8–133.7, *I*^2^ = 100%).

A *post-hoc* sensitivity analysis showed that the admission rate was 131.6 per 1000 live births (95% CI = 121.9–141.3) when using the originally reported live-birth denominator. When the denominator was adjusted to account for potential underreporting through a stillbirth correction, the estimated rate increased to 154.2 per 1000 live births (95% CI = 133.4–175.1) (Figure S8 in the **Online Supplementary Document**).

Our analysis indicated the presence of publication bias (Egger’s test *P*-value <0.001). The pooled neonatal admissions in studies with a low risk of bias were 147.1 (95% CI = 137.2–157.0, *I*^2^ = 100%), while those in studies with a high risk of bias were 75.3 (95% CI = 39.7–110.9, *I*^2^ = 99.9%) (Figure S6 and Table S10 in the **Online Supplementary Document**).

The residual between-study variance (*τ*^2^) in the meta-regression analysis across all five models was reduced to negligible levels, indicating that the included covariates accounted for nearly all observable heterogeneity in effect sizes. For models incorporating setting, income group, NMR, and WHO region as covariates, *τ*^2^ estimates ranged from 5.1 × 10^−6^ to 8.7 × 10^−6^. Even the model based on level of care (secondary *vs*. tertiary), which had the highest residual variance (*τ*^2^ = 0.005), showed only minor unexplained dispersion. The low *τ*^2^ across models suggests that variation in neonatal admissions per 1000 live births was largely explained by these factors, with little residual heterogeneity remaining after adjustment.

### Length of stay

**Figure 3:**
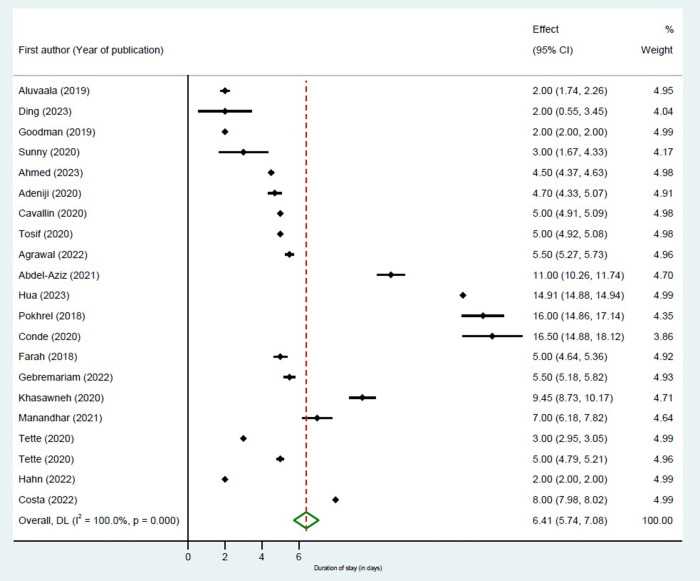
Overall length of hospital stay.

The estimated average duration of stay was 6.4 days (95% CI = 5.7–7.1, *I*^2^ = 100%, 21 estimates from 20 studies, very low certainty of evidence) (**Figure 3**, **Table 2**; Table S12 in the **Online Supplementary Document**). Our analysis did not reveal any publication bias (Egger’s test *P*-value = 0.07). In a *post-hoc* analysis based on the study reported outcome measure (mean, median, proportion), we found that the pooled length of stay was 14.18 days (95% CI = 11.11–17.24, *I*^2^ = 98.7%, four studies) where mean duration was reported, while it was 3.58 days (95% CI = 3.44–3.71, *I*^2^ = 99.9%, 11 studies) where median duration was reported, and 6.57 days (95% CI = 4.77–8.36, *I*^2^ = 99.9%, six studies) where length of stay was expressed as a proportion. The average duration of stay varied between 7.1 days (95% CI = 4.2–9.9, *I*^2^ = 100%,16 estimates from 15 studies) in studies with low risk of bias and 4.8 days (95% CI = 3.1–6.5, *I*^2^ = 99.8%; five studies) in studies with high risk of bias (Figure S7 and Table S11 in the **Online Supplementary Document**). The subgroup analysis of the length of stay by the level of care indicated that the average length of stay was 6.8 days (95% CI = 5.02–8.65, *I*^2^ = 100%) at the tertiary level and 4.1 days (95% CI = 2.2–6.1, *I*^2^ = 88.5%) at the secondary level of care.

### Number of beds required per 1000 live births

Based on pooled neonatal admissions and length of stay, the estimated number of beds necessary for the care of SSNs was 2.4 (95% CI = 2.0–2.8) per 1000 live births (**Table 2**).

Based on region-specific rates for neonatal admissions and length of stay, the estimated bed requirement for SSN care per 1000 live births was 1.9 in the African region and 2.6 in Southeast Asia (**Table 3**). The Region of the Americas had the lowest number of beds needed at 1.7, while the Eastern Mediterranean region had the highest requirement at 4.0 per 1000 live births.

**Table 3:** Number of beds for care of SSNs per 1000 LBs, by WHO region

WHO region	Admission	Length of stay*	Beds
Africa	160.5	4.3	1.9
Western Pacific	151.4	5.0	2.1
Eastern Mediterranean	143.0	10.2	4.0
Region of the Americas	122.2	5.2	1.7
South-East Asia	121.5	7.9	2.6
Europe	96.5	14.9	3.9

WHO – World Health Organization

*Used the corresponding region-wise length of stay.

## DISCUSSION

Our analysis indicates that the pooled estimate for neonatal admissions was 134.6 per 1000 live births, though this varied substantially by region and income level. Notably, most of the included studies (30 out of 34) were conducted in tertiary care settings, which may have led to an over-representation of more severe cases. Higher admission rates were observed in the low-income settings within the African and Western Pacific regions. The average length of stay was estimated to be 6.4 days (95% CI = 5.7–7.1), with significant heterogeneity across settings. Based on these parameters, we estimated that 2.4 beds (95% CI = 2.0–2.8) per 1000 live births are needed for SSN care, with the highest bed requirement observed in the Eastern Mediterranean region.

The variation in bed requirements for SSN care across regions can be attributed to differing norms for neonatal admissions and hospital stay durations. Our analysis suggests that the number of neonates requiring admission ranged from 96 to 160 per 1000 live births in various WHO regions. Hospital policies regarding mandatory NICU admissions may vary by region, both within and among countries, based on gestational age or birth weight thresholds [69,70]. In California, Schulman and colleagues demonstrated a 34-fold variation in inborn NICU admission rates for infants ≥34 weeks of gestation [71]. This variation was not consistently associated with the level of care or the proportion of admissions involving higher illness acuity. Another study within the Vermont Oxford Network noted a 30-fold difference in NICU admission rates for late preterm, early term, and term infants across various NICU levels and within NICUs of the same level [72]. The Dartmouth Atlas of Neonatal Intensive Care highlighted geographic variation in NICU admissions based on birth weight [73]. While there was a slight variation for infants weighing between 500 g and 1499 g, higher birth weight categories exhibited a three- to five-fold increase in admission rates in certain regions. Goodman and colleagues identified geographical and hospital disparities in late preterm admission rates among infants insured by Texas Medicaid, even after adjusting for newborn health risks [26]. An evaluation of level 2 neonatal care in low- and middle-income countries such as Uganda, Indonesia, and India revealed significant variability in facility readiness, record maintenance, and limitations in the availability of appropriate equipment and trained personnel. In hospitals that maintain birth weight records, more than half of the admitted newborns weighed ≥2500 g [74].

The region-specific neonatal admission rates observed in our analysis align with the underlying burden of neonatal morbidity and mortality reported in those regions. We found the highest admission rates per 1000 live births in Africa (160.5) and the lowest in Europe (96.5). Prior studies have similarly documented a high burden of neonatal disorders in sub-Saharan Africa, with an age-standardised incidence rate of 432.4 per 100 000 (95% uncertainty interval (UI) = 402.9–469.5), compared to substantially lower rates in Western Europe (176.5; 95% UI = 171.8–181.4) [75]. Neonatal mortality shows a similar pattern, with sub-Saharan Africa reporting 50.2 deaths per 1000 live births (95% UI = 41.9–60.9) and Western Europe reporting only 3.1 (95% UI = 2.6–3.7) [76]. Consistent with these trends, our study found that settings with an NMR above 12 per 1000 live births had higher admission rates (149.4) compared to those with lower NMRs (126.3). These findings suggest that regions with greater neonatal morbidity and mortality are associated with increased demand for facility-based care, as reflected in higher admission rates.

Our estimate of beds required per 1000 live births is driven by two key factors: the neonatal admission rate and the average length of hospital stay. Notably, the Eastern Mediterranean (NMR = 12.2) and European regions (NMR = 5.8) showed similarly high bed requirements (~4 beds per 1000 live births), despite differing mortality profiles. This reflects a trade-off between high admission rates and shorter stays in the Eastern Mediterranean vs lower admissions and longer stays in Europe. The relationship between length of stay and neonatal mortality is complex, influenced by healthcare practices, resource availability, and case mix [77]. For instance, improved survival of extremely preterm infants and advanced neonatal care in high-income settings often prolong hospitalisation [78,79]. Moreover, NICU bed availability itself can influence discharge timing, with lower census linked to longer stays [80].

Determining the number of neonatal beds needed across various WHO regions is a complex process involving multiple factors related to neonatal health morbidities and their severity, mortality rates, and length of stay. It is also influenced by local policy, healthcare infrastructure capacity, and available referral systems. Our estimates based on admission may not accurately reflect the true need and should be interpreted with caution. For instance, NICUs from the European region seem to require more beds because improving care allows a growing proportion of extremely preterm infants to survive and occupy intensive care for longer periods [81]. Demand is further amplified by caring for infants with congenital anomalies, which account for a disproportionately high share of neonatal hospital days and surgical episodes [82]. By contrast, in many low-income countries, including those in Africa, infectious conditions like neonatal sepsis remain the leading cause of admission. Limited resources, delayed care-seeking, combined with persistently high NMRs, often result in shorter reported length of stay, contributing to a lower estimated number of required beds even though the underlying unmet need is greater [83]. In these situations, population incidence-based methods may provide better estimates of the beds required, provided granular data are available.

Beyond routine service provision, unexpected events such as disease outbreaks or natural disasters can significantly increase the demand for neonatal beds. While our estimate of 2.4 beds per 1000 live births (95% CI = 2.0–2.8) provides a standardised baseline for planning, regional decision-making should incorporate local epidemiological data and health system dynamics, including neonatal morbidity patterns, institutional delivery rates, referral systems, and care-seeking behaviours. To ensure adequate surge capacity and account for operational realities such as delays in discharge, evolving causes of admission, and variability in daily occupancy, it is prudent to plan for an 85% bed occupancy rate, allowing for a 15% buffer [84,85]. Applying this adjustment increases the overall estimated requirement to 2.8 beds per 1000 live births (95% CI = 2.3–3.22) for SSN care. Moreover, as geographic units typically have more than 1000 live births, this estimate should be scaled accordingly. For instance, in a setting with 5000 live births, the adjusted requirement would be approximately 14 beds (95% CI = 11.5–16.1). This approach supports more resilient planning, ensuring sufficient capacity to meet both routine and surge demand for small and/or sick newborn care.

This study has several limitations. First, we acknowledge that estimating bed requirements based on current admission rates may underestimate the actual demand. Ideally, bed projections should be based on the population-level burden of severe neonatal morbidities that require hospital care, as these reflect the number of neonates needing care rather than just those who can access hospital services. Although we attempted to estimate the number of neonates requiring admission using Global Burden of Disease data, we encountered significant limitations due to the lack of detailed data on disease severity, particularly regarding coexisting or multiple morbidities (*e.g.* neonates with both prematurity and sepsis). Without detailed data on multimorbidity, such estimates risk double-counting cases and artificially inflating projected bed needs. The issue of double counting is less relevant in hospital admission estimates because multiple conditions in a single infant would still be recorded as just one admission. While our approach based on observed admissions provides a reliable estimate of current utilisation, it likely understates the true need, particularly for critically ill neonates who never reach a facility. Community-based studies show that only about 20% of sick neonates receive care at health facilities [86]. Additionally, assessments of facility readiness in many low- and middle-income countries reveal ongoing deficiencies in neonatal intensive care capacity [74]. In settings with limited referral systems, poor access to care, or high rates of home births, estimates can be adjusted using a correction factor derived from the inverse of hospital live birth reporting rates specific to the region [87]. Second, most (88.2%) of the included studies were hospital-based, particularly from tertiary-level facilities, indicating that the estimation may be biased towards severe cases requiring specialised care. Third, the available data were insufficient to estimate admission rates and bed requirements for specific neonatal subgroups, such as those born preterm, with low birth weight, or small for gestational age.

## CONCLUSIONS

This data-driven method quantifies the estimated number of beds required for the care of sick and small newborns by employing a systematic approach that incorporates available data on neonatal admission rates and length of stay. These findings offer a useful starting point for healthcare resource planning. Further refinement of these estimates is necessary as more data on neonatal morbidities become available. Local adaptation of these estimates using region-specific morbidity profiles will be critical to inform policy and ensure adequate infrastructure for the care of sick and small newborns.

## Additional material


Online Supplementary Document
