# Supplementary material for: Estimating the number of hospital beds for the care of sick and small newborns: an evidence-based systematic approach
Source: J Glob Health. 2025 Oct 14;15:04312. doi: 10.7189/jogh.15.04312 (PMC12517701; doi:10.7189/jogh.15.04312)
Supplement: Online Supplementary Document [file jogh-15-04312-s001.pdf]

Supplement to: Sinha B, Kumar M, Thomas D, Strobel N, Gupta G, Edmond K, Sankar MJ. Estimating the number of hospital beds for the care of sick and small newborns: an evidence-based systematic approach. J Glob Health. 2025;15:04312.

Title: Estimating the number of beds for care of sick and small newborns: An evidence based systematic approach

Running title: The number of beds used for sick and small newborns

Author Names

Bireshwar Sinha[1], Mohan Kumar[2], Deena Thomas[3], Natalie Strobel[4], Gagan Gupta[5], Karen Edmond[6], Mari Jeeva Sankar[7]

Online Supplementary Document

Table S1: Detailed search strategy by database

Table S2: Detailed characteristics of included studies

Table S3: Detailed characteristics of studies found eligible but excluded

Table S4: Risk of bias assessment using JBI critical appraisal tool

Table S5: Number of neonatal admissions per 1000 live births, by WHO region

Figure S1: Number of neonatal admissions per 1000 live births, by WHO region

Table S6: Number of neonatal admissions per 1000 live births, by income status

Figure S2: Number of neonatal admissions per 1000 live births, by income status

Table S7: Number of neonatal admissions per 1000 live births, by study setting

Figure S3: Number of neonatal admissions per 1000 live births, by study setting

Table S8: Number of neonatal admissions per 1000 live births, by level of health facility

Figure S4: Number of neonatal admissions per 1000 live births, by level of health facility

Table S9: Number of neonatal admissions per 1000 live births, by neonatal mortality rate (NMR) category

Figure S5: Number of neonatal admissions per 1000 live births, by neonatal mortality rate (NMR) category

Table S10: Number of neonatal admissions per 1000 live births, by risk of bias

Figure S6: Number of neonatal admissions per 1000 live births, by risk of bias

Table S11: Subgroup analysis of duration of stay by risk of bias

Figure S7: Subgroup analysis of duration of stay by risk of bias

Figure S8: Number of neonatal admissions per 1000 live births, by denominator (study specified or data derived using study duration/settings specific stillbirth rate)

Table S12: GRADE assessment

Table S1: Detailed search strategy to estimate the number of neonatal admissions to the SNCUs or the NICUs in any setting

Embase *via Ovid* <1974 to 2023 May 01>

#	**Search**
1	epidemiology/
2	exp case control study/
3	cohort analysis/
4	cross-sectional study/
5	case study/
6	longitudinal study/
7	observational study/
8	correlational study/
9	"population research"/
10	epidemiologic.ab,ti.
11	case control.ab,ti.
12	case referent.ab,ti.
13	'case stud$3'.ab,ti.
14	case series.ab,ti.
15	cohort$1.ab,ti.
16	cross sectional.ab,ti.
17	observational.ab,ti.
18	1 or 2 or 3 or 4 or 5 or 6 or 7 or 8 or 9 or 10 or 11 or 12 or 13 or 14 or 15 or 16 or 17
19	(infan$3 or (newborn or new born or newly born) or (neonat$2 or "neo nat$2") or (baby or babies) or perinatal).ti,ab,kw.
20	perinatal period/ or perinatal care/
21	infant/ or baby/ or newborn/
22	19 or 20 or 21
23	hospital admission/
24	incidence/ or cumulative incidence/

#	Search
25	prevalence/ or period prevalence/ or point prevalence/
26	"Admissions".kw.
27	((admission$1 adj3 rate$1) or (admission$1 adj3 hospital$7)).ti,ab.
28	(hospitali#ation$1 adj3 rate$1).ti,ab.
29	((length adj2 stay) or "length of stay" or ((extended or long or short or brief) adj3 (admission$1 or hospitali#ation$1))).ti,ab.
30	((all adj2 cause$1) or "all-cause$1" or "all cause$1").ti,ab.
31	(duration adj2 (admit$3 or admission$1)).ti,ab.
32	((episod$2 adj3 care) or (hospital$7 adj3 episod$2)).ti,ab.
33	(incidence$1 adj2 rate$1).ti,ab.
34	(prevalence$1 adj2 rate$1).ti,ab.
35	23 or 24 or 25 or 26 or 27 or 28 or 29 or 30 or 31 or 32 or 33 or 34
36	18 and 22 and 35
37	coronavirus disease 2019/ or (COVID-19 or covid$9 or influenza).ti,ab,kw.
38	"review"/ or "systematic review"/ or scoping review.ti,ab,kw.
39	("Randomi#ed control$3" or "double blind" or (phase adj (I or II or III or IV)) or (clinical adj (effectiveness or outcome or assessment))).ti,ab,kw.
40	(rat or rats or mouse or mice or rodent or rodents or swine or porcine or murine or sheep or lamb or lambs or ewe or ewes or pig or pigs or piglet or piglets or sow or sows or rabbit or rabbits or cat or cats or kitten or kittens or dog or dogs or puppy or puppies or monkey or monkeys or horse or horses or foal or foals or equine or calf or calves or cattle or heifer or heifers or hamster or hamsters or chicken or chickens or livestock or panda or pandas or buffalo$ or baboon$ or nonhuman or non-human).mp.
41	exp "meta analysis (topic)"/ or meta analysis/ or network meta-analysis/ or exp "review"/ or (systematic adj (review$1 or overview$1)).ti,ab,kw. or "literature review".ti,ab,kw. or ("meta analy$3" or "metaanaly$3" or "meta-analy$3").ti,ab,kw.
42	((maternal adj2 (morbidity or mortality or complication or outcomes)) or cancer$1 or diabet$2 or asthma or obesity or dental or anxiety or depression or "mental illness" or (breastfeeding adj2 outcome$1) or adult$1 or aged or preeclampsia or pre-eclampsia).ti,ab,kw.
43	36 not (37 or 38 or 39 or 40 or 41 or 42)

#	Search
44	limit 43 to yr="2018 -Current"

MEDLINE(R) ALL *via Ovid* <1946 to May 01, 2023>

#	Search
1	Epidemiology/
2	exp case-control studies/
3	cohort analysis/
4	Cross-Sectional Studies/
5	Case Reports/
6	Longitudinal Studies/
7	observational study/
8	epidemiologic studies/
9	"Health services research".kw.
10	epidemiologic.ab,ti.
11	case control.ab,ti.
12	case referent.ab,ti.
13	"case stud$3".ab,ti.
14	case series.ab,ti.
15	cohort$1.ab,ti.
16	cross sectional.ab,ti.
17	observational.ab,ti.
18	1 or 2 or 3 or 4 or 5 or 6 or 7 or 8 or 9 or 10 or 11 or 12 or 13 or 14 or 15 or 16 or 17
19	(infan$3 or (newborn or new born or newly born) or (neonat$2 or "neo nat$2") or (baby or babies) or perinatal).ti,ab,kf.
20	Perinatal Care/
21	infant/ or exp Infant, Newborn/
22	19 or 20 or 21
23	"episode of care"/ or hospitalization/ or "length of stay"/ or patient admission/
24	incidence/
25	prevalence/
26	"hospital admissions".kw.

#	Search
27	((admission$1 adj3 rate$1) or (admission$1 adj3 hospital$7)).ti,ab.
28	(hospitali#ation$1 adj3 rate$1).ti,ab.
29	((length adj2 stay) or "length of stay" or ((extended or long or short or brief) adj3 (admission$1 or hospitali#ation$1))).ti,ab.
30	((all adj2 cause$1) or "all-cause$1" or "all cause$1").ti,ab.
31	(duration adj2 (admit$3 or admission$1)).ti,ab.
32	((episod$2 adj3 care) or (hospital$7 adj3 episod$2)).ti,ab.
33	(incidence$1 adj2 rate$1).ti,ab.
34	(prevalence$1 adj2 rate$1).ti,ab.
35	23 or 24 or 25 or 26 or 27 or 28 or 29 or 30 or 31 or 32 or 33 or 34
36	18 and 22 and 35
37	covid-19/ or post-acute covid-19 syndrome/ or (COVID-19 or covid$9 or influenza).ti,ab,kf.
38	"review"/ or "systematic review"/ or scoping review.ti,ab,kf.
39	("Randomi#ed control$3" or "double blind" or (phase adj (I or II or III or IV)) or (clinical adj (effectiveness or outcome or assessment))).ti,ab,kf.
40	(rat or rats or mouse or mice or rodent or rodents or swine or porcine or murine or sheep or lamb or lambs or ewe or ewes or pig or pigs or piglet or piglets or sow or sows or rabbit or rabbits or cat or cats or kitten or kittens or dog or dogs or puppy or puppies or monkey or monkeys or horse or horses or foal or foals or equine or calf or calves or cattle or heifer or heifers or hamster or hamsters or chicken or chickens or livestock or panda or pandas or buffalo$ or baboon$ or nonhuman or non-human).mp.
41	"review"/ or meta-analysis/ or "systematic review"/ or (systematic adj (review$1 or overview$1)).ti,ab,kf. or "literature review".ti,ab,kf. or ("meta analy$3" or "metaanaly$3" or "meta-analy$3").ti,ab,kf.
42	((maternal adj2 (morbidity or mortality or complication or outcomes)) or cancer$1 or diabet$2 or asthma or obesity or dental or anxiety or depression or "mental illness" or (breastfeeding adj2 outcome$1) or adult$1 or aged or preeclampsia or pre-eclampsia).ti,ab,kf.
43	36 not (37 or 38 or 39 or 40 or 41 or 42)
44	limit 43 to yr="2018 -Current"

Cochrane CENTRAL *via Cochrane Library* | Date Run: 03/05/2023

ID	Search	Hits
#1	(infan* or newborn or (new born) or (newly born) or neonat* or (neo nat*) or baby or babies):ti,ab,kw	91190
#2	MeSH descriptor: [Infant, Newborn] explode all trees	20430
#3	#1 OR #2	91190
#4	((admission* NEAR/3 rate*) OR (admission* NEAR/3 hospital)):ti,ab,kw	14998
#5	MeSH descriptor: [Patient Admission] this term only	728
#6	MeSH descriptor: [Incidence] this term only	15051
#7	MeSH descriptor: [Prevalence] this term only	8684
#8	(hospitali?ation NEAR/3 rate*):ti,ab,kw	3099
#9	MeSH descriptor: [Hospitalization] this term only	10609
#10	((length NEAR/2 stay) or ("length of stay") or ((extended or long or short or brief) NEAR/3 (admission* or hospitali?ation*))):ti,ab,kw	25227
#11	((all NEAR/2 cause*) or (all-cause) or (all cause)):ti,ab,kw	37013
#12	(duration NEAR/2 (admit* or admission*)):ti,ab,kw	372
#13	((episode* NEAR/3 care) or (hospital* NEAR/3 episode*)):ti,ab,kw	963
#14	MeSH descriptor: [Patient Readmission] this term only	1378
#15	#4 OR #5 OR #6 OR #7 OR #8 OR #9 OR #10 OR #11 OR #12 OR #13 OR #14	104102
#16	MeSH descriptor: [Epidemiologic Studies] explode all trees	193601
#17	MeSH descriptor: [Observational Studies as Topic] this term only	210
#18	MeSH descriptor: [Observational Study] explode all trees	1537
#19	MeSH descriptor: [Cohort Studies] this term only	10360
#20	MeSH descriptor: [Validation Studies as Topic] this term only	44
#21	MeSH descriptor: [Cross-Sectional Studies] this term only	8168
#22	((observation* NEAR/3 (stud* or design or analys*)) or (cohort* NEAR/3 (stud* or design or analys*)) or ((longitudinal or longterm or (long NEXT term)) NEAR/3 (stud* or design or analys*)) or ((case NEXT control) or (case NEXT comparison) or (case NEXT controlled)) or (case-referent NEAR/3 (stud* or design or analys*)) or (population NEAR/3 (stud* or design or analys*)) or (descriptive near/3 (stud* or design or analys*)) or	208550

	((multidimensional or (multi next dimensional)) near/3 (stud* or design or analys*)) or (cross NEXT sectional) or (natural NEXT experiment*) or (quasi NEXT experiment*) or ((non experiment or nonexperiment or non experimental or nonexperimental) NEAR/3 (stud* or design or analys*)) or (ecologic* NEAR/3 (stud* or design or analys*))):ti,ab,kw	
#23	#16 OR #17 OR #17 OR #18 OR #19 OR #20 OR #21 OR #22	344415
#24	#3 AND #15 AND #23	2872
#25	(rat or rats or mouse or mice or rodent or rodents or swine or porcine or murine or sheep or lamb or lambs or ewe or ewes or pig or pigs or piglet or piglets or sow or sows or rabbit or rabbits or cat or cats or kitten or kittens or dog or dogs or puppy or puppies or monkey or monkeys or horse or horses or foal or foals or equine or calf or calves or cattle or heifer or heifers or hamster or hamsters or chicken or chickens or livestock or panda or pandas or buffalo* or baboon* or nonhuman or non-human):ti,ab,kw	58426
#26	MeSH descriptor: [COVID-19] explode all trees	4255
#27	(COVID-19 or covid* or influenza):ti,ab,kw	24237
#28	MeSH descriptor: [Meta-Analysis as Topic] explode all trees	1447
#29	(meta analy* or metaanaly* or meta-analy* or (systematic next (review* or overview*))):ti,ab,kw	26607
#30	MeSH descriptor: [Review Literature as Topic] explode all trees	428
#31	#28 OR #29 OR #30	26739
#32	MeSH descriptor: [Randomized Controlled Trial] explode all trees	25733
#33	MeSH descriptor: [Randomized Controlled Trials as Topic] explode all trees	47399
#34	(maternal NEAR/2 (morbidity or mortality or complication* or outcome*))	5009
#35	(cancer* or diabet* or asthma or obesity or dental or anxiety or depression or (mental illness) or (breastfeeding NEAR/2 outcome*) or adult* or aged or preeclampsia or pre-eclampsia):ti,ab,kw	1239714
#36	#24 NOT #25	2703
#37	#36 NOT #26	2695
#38	#37 NOT #27	2650

#39	#38 NOT #31	2514
#40	#39 NOT #32	2473
#41	#40 NOT #33	2359
#42	#41 NOT #34	2282
#43	#42 NOT #35	1186
#44	#42 NOT #35<br>with Cochrane Library publication date Between Jan 2018 and Dec 2023	354

Table S2: Detailed characteristics of included studies

Study No	Study author, year	Country; study period	Study type and setting	Level of health facility	Comments
	**Number of neonates admitted**				
1.	**Abdellatif, 2019**[1]	Oman; Jan 2007-Dec 2014	Cross-sectional hospital based	Tertiary	Both number of admissions and live births provided. duration of stay not provided
2.	**Agrawal,2022**[2]	India; Jan- Dec 2021	Retrospective hospital based observational	Tertiary	Both number of admissions and live births provided. duration of stay provided as proportion. Newborns admitted to paediatric ward/PICU were excluded
3.	**Ahmed,2023**[3]	Ethiopia; May 2019-May 2021	Retrospective hospital based cohort	Tertiary	Both number of admissions and births provided. Live births calculated after taking country wise still birth rate into account. Duration of stay provided as proportion
4.	**Braun,2020**[4]	US; Jan 2010- Dec 2018 (2018 data taken)	Retrospective hospital based cohort	Tertiary	Both number of admissions and live births provided. Duration of stay provided as proportion (<3 or >3 days from which median/mean could not be calculated)
5.	**Cavallin, 2020**[5]	Ethiopia; Jan 2014-Dec 2017	Retrospective hospital based observational	Tertiary	Both number of admissions and births provided. Live births calculated after taking country wise still birth rate into account. Duration of stay provided.

6.	**Choi, 2019[(6)]**	Rawanda; July 2011- Sep 2017	Cross-sectional hospital based	Tertiary	Both number of admissions and live births provided. Duration of stay not provided
7.	**Conde, 2020[(7)]**	Mexico; 2016-2019	Hospital based case series	Tertiary	Both number of admissions and live births provided. Duration of stay provided
8.	**Dumpa,2019[(8)]**	US; 2011-2014	Retrospective population based	NA	Both number of admissions and live births provided. Duration of stay not provided
9.	**Eftekharian,2021[(9)]**	Austria; Jan 2003- Dec 2013	Retrospective hospital based observational	Tertiary	Both number of admissions and live births provided. Congenital malformation and incomplete records removed. Duration of stay not provided
10.	**Faulks,2023[(10)]**	Australia; 1999-2016	Retrospective population based	NA	Both number of admissions and live births provided. Congenital anomalies excluded. Duration of stay not provided
11.	**Fradkin,2022[(11)]**	US; Jan 2007- Dec 2016	Retrospective hospital based observational	Tertiary	Both number of admissions and live births provided. Duration of stay not provided.
12.	**Goodman,2019[(12)]**	US; 2010-2014	Retrospective population based	NA	Both number of admissions and births provided. Live births calculated after taking country wise still birth rate into account. Duration of stay provided.
13.	**Guidozzi,2018[(13)]**	South Africa; Sep 2016- Oct 2016	Retrospective hospital based observational	Tertiary	Both number of admissions and live births provided. Duration of stay not provided. Congenital anomalies and missing data excluded.

14.	**Hahn, 2022**[14]	US; Jan 2016- Dec 2020	Retrospective hospital based observational	Tertiary	Both number of admissions and live births provided. Duration of stay not provided.
15.	**Harrison, 2018**[15]	Us; Jan 2013- Dec 2013	Retrospective population based	NA	Both number of admissions and live births provided. Duration of stay not provided.
16.	**Herbozo,2021**[16]	Peru; Jan 2015- Dec 2019	Retrospective hospital based observational	Tertiary	Both number of admissions and live births provided. Duration of stay not provided.
17.	**Howell, 2020**[17]	US; 2014-2015	Retrospective population based; secondary data	NA	Both number of admissions and live births provided. Average length of delivery stay (days) was 12.3 among twins and 4.1 among singletons.
18.	**Hua, 2023** [18]	England; Jan 2005- Dec 2006	Retrospective population based	NA	Both number of admissions and live births provided. Duration of stay provided.
19.	**Jones, 2018** [19]	England; April 2008- March 2014	Retrospective population based	NA	Both number of admissions and live births provided. Overall duration of stay provided. Morbidity wise duration of stay provided.
20.	**Juliana, 2022**[20]	Suriname; May 2017- Dec 2018	Prospective observational population based study	NA	Though a hospital based study; it has been considered population based as it was the only NICU in the region. Both number of admissions and live births provided. Duration of stay not provided.

21.	**Khasawneh,2020**[21]	Jordan; Sep 2016- Sep 2018	Retrospective cross-sectional hospital based	Tertiary	Both number of admissions and live births provided. Duration of stay not provided.
22.	**Kim,2021**[22]	US; Jan 2008- Dec 2018	Retrospective population based cohort	NA	Both number of admissions and live births provided. Duration of stay not provided.
23.	**Lanier,2021**[23]	US; April 2015- Dec 2019	Prospective observational hospital based study	Tertiary	Both number of admissions and live births provided. Duration of stay not provided.
24.	**Li,2019**[24]	China; May 2015- Aug 2017	Prospective observational hospital based study	Tertiary	Both number of admissions and live births provided. Duration of stay not provided.
25.	**Li,2020**[25]	Multicentric: South east Asian and African region; Jun 2017- May 2018	Prospective observational hospital based study	Tertiary	Both number of admissions and live births provided. Duration of stay not provided. Neonatal AMR study network.
26.	**Lundeby, 2020**[26]	Somalia; Jun 2013- Oct 2013	Retrospective hospital based observational	Tertiary	Both number of admissions and live births provided. Duration of stay not provided.

27.	**Manandhar,2021**[27]	Nepal; April 2016- Oct 2017	Prospective observational hospital based study	Tertiary	Both number of admissions and live births provided. Duration of stay provided.
28.	**Massad,2020** [28]	Palestine; Jun 2016- August 2018	Cross sectional hospital based study	Tertiary	Both number of admissions and births provided. Live births calculated after taking country wise still birth rate into account. Duration of stay not provided.
29.	**Mdoe,2022**[29]	Tanzania; Jan 2019- May 2020	Cross sectional hospital based study	Tertiary	Both number of admissions and live births provided. Duration of stay not provided.
30.	**Moshiro, 2019**[30]	Tanzania; Oct 2014- July 2017	Prospective observational hospital based study	Tertiary	Both number of admissions and live births provided. Duration of stay not provided.
31.	**Moura, 2020**[31]	Brazil; July 2012- Dec 2012	Hospital based cohort	Tertiary	Both number of admissions and live births provided. Duration of stay not provided.
32.	**Murphy,2018**[32]	Kenya; July 2014- April 2016	Cross-sectional plus retrospective review of admission events, hospital based	Tertiary	Both number of admissions and live births provided. Duration of stay not provided.

33.	**Pokhrel,2018**[33]	Nepal; April 2014- April 2017	Retrospective hospital based cross sectional	Tertiary	Both number of admissions and live births provided. Duration of stay provided.
34.	**Reyesa, 2018**[34]	Mexico; Jan 2016- Jan 2017	Prospective hospital based cohort	Tertiary	Both number of admissions and live births provided. Duration of stay not provided.
35.	**Sackey, 2019**[35]	Ghana; 2011-2015 (2015 data taken)	Retrospective hospital based observational	Tertiary	Both number of admissions and births provided. Live births calculated after taking country wise still birth rate into account. Duration of stay not provided
36.	**Seeni, 2019**[36]	US; 2002- 2008	Retrospective hospital based cohort	Tertiary	Both number of admissions and births provided. Singleton births were given which was taken as liver births. Duration of stay not provided.
37.	**Seid, 2019**[37]	Ethiopia; Sep 2014- Sep 2017	Retrospective hospital based cross sectional	Tertiary	Both number of admissions and live births provided. Duration of stay provided.
38.	**Serra, 2019**[38]	Italy; Jan 2017-Dec 2018	Retrospective hospital based cohort	Primary	Both number of admissions and live births provided. Duration of stay not provided.
39.	**Sunny, 2020** [39]	Nepal; March 2015- April 2015	Hospital based cross sectional	Secondary	Both number of admissions and live births provided. Duration of stay provided as proportion

40.	**Tadesse, 2021** [40]	Ethiopia; Jun 2016- March 2019	Retrospective hospital based cohort	Tertiary	Both number of admissions and births provided. Live births calculated after taking country wise still birth rate into account. Duration of stay not provided
41.	**Tosif, 2018** [41]	Solomon islands; 2014	Hospital based cross sectional	Secondary	Both number of admissions and live births provided. Duration of stay not provided.
42.	**Tosif, 2020** [42]	Solomon islands; Jan 2014- Dec 2016	Retrospective hospital based cohort	Secondary	Both number of admissions and live births provided. Duration of stay provided.
43.	**Wehrmeister, 2019** [43]	Brazil: Jan 2015- Dec 2015	Retrospective population based cohort	NA	Both number of admissions and live births provided. Duration of stay not provided.
44.	**Xu, 2022** [44]	China; 2015	Retrospective population based cohort	NA	Both number of admissions and live births provided. Duration of stay not provided.
45.	**Zafeiri, 2022** [45]	UK; 1985- 2015	Retrospective population based cohort	NA	Both number of admissions and live births provided. Duration of stay not provided.
46.	**Ziem, 2023** [46]	Ghana; Jan 2021- Dec 2021	Retrospective hospital based cross sectional	Tertiary	Both number of admissions and live births provided. Duration of stay not provided.
	**Duration of stay: Studies included: 2, 3, 5, 7, 12, 14, 18, 21, 27, 33, 39, 42**				

1.	**Abdel-Aziz, 2021**[47]	Egypt; Jan 2020-Dec 2020	Prospective hospital based observational	Tertiary	Duration of stay provided as proportion
2.	**Adeniji, 2020** [48]	Nigeria; Jan 2017-Jun 2017	Hospital based cross sectional	Tertiary	Duration of stay provided
3.	**Aluvaala, 2019**[49]	Kenya; April 2014-Dec 2015	Retrospective hospital based cohort	Tertiary	Duration of stay provided
4.	**Costa, 2020**[50]	Brazil; Jan 2013-Dec 2015	Hospital based cross sectional	Tertiary	Duration of stay provided
5.	**Ding, 2023**[51]	US; 2019	Population based cross sectional	NA	Duration of stay provided
6.	**Farah, 2018**[52]	Ethiopia; May 2017- Jun 2017	Retrospective hospital based	Tertiary	Duration of stay provided as proportion
7.	**Gebremariam, 2022**[53]	Eritrea; Jan 2018-Dec 2021	Retrospective hospital based	Tertiary	Duration of stay provided as proportion
8.	**Tette, 2020**[54]	Ethiopia; Sep 2020- Dec 2020	Cross sectional hospital based	Tertiary	Duration of stay provided as proportion

Table S3: Detailed characteristics of studies found eligible but excluded

Author Name	Reasons for exclusion
**Al-Shehri**[55]	The type of study was not clear; use of ecological data
**Bajaj** [56]	The denominator – population covered/number of births or live births and duration of stay was not provided
**Eshete** [57]	The denominator – population covered/number of births or live births and duration of stay was not provided
**Hadgu** [58]	The denominator – population covered/number of births or live births – was not provided. The data on length of hospital stay was provided in predefined categories of <7 days and ≥7 days – median (IQR) could not be computed.
**Hanson** [59]	The denominator – population covered/number of births or live births and duration of stay was not provided
**Menalu** [60]	No data on number of admissions and duration of hospital stay
**Mengistu** [61]	The denominator – population covered/number of births or live births and duration of stay was not provided
**Merscher** [62]	The denominator – population covered/number of births or live births and duration of stay was not provided
**Meshram** [63]	The denominator – population covered/number of births or live births and duration of stay was not provided
**Moise** [64]	The denominator – population covered/number of births or live births and duration of stay was not provided

**Mokuolu** (65)	The denominator – population covered/number of births or live births and duration of stay was not provided
**Monoghan** (66)	The denominator – population covered/number of births or live births and duration of stay was not provided
**Nyishime** (67)	The denominator – population covered/number of births or live births and duration of stay was not provided
**Ojima** (68)	The denominator – population covered/number of births or live births and duration of stay was not provided
**Opare-Asamoah** (69)	The denominator – population covered/number of births or live births and duration of stay was not provided
**Orsido** (70)	The denominator – population covered/number of births or live births and duration of stay was not provided
**Srivastava** (71)	The denominator – population covered/number of births or live births and duration of stay was not provided
**Tolossa** (72)	The denominator – population covered/number of births or live births and duration of stay was not provided

JBI critical appraisal tool used for risk of bias analysis contains following 9 parameters

1. Sample frame appropriate?
2. Sampling appropriate?
3. Sample size adequate?
4. Study subjects and settings described in detail?
5. Sufficient coverage?
6. Valid methods used for identification of condition?
7. Condition measured in standard way for all?
8. Population adjusted?
9. Response rate adequate?

Table S4: Risk of bias analysis using JBI critical appraisal tool of all the included studies

First author	Year of publication	Covidence #	1	2	3	4	5	6	7	8	9	Final ROB
Abdel-Aziz	2021	23788	Yes	Yes	Yes	Yes	Yes	Unclear	Yes	Yes	Yes	Low
Abdellatif	2019	23793	Yes	Yes	Yes	Yes	Yes	Yes	Yes	Yes	Unclear	Low
Adeniji	2020	23907	Yes	Yes	Yes	Yes	No	Yes	Yes	Yes	Yes	Low
Agrawal	2022	23977	Yes	Yes	Yes	Yes	Yes	Yes	Unclear	Yes	Yes	Low
Ahmed	2023	24011	Yes	Yes	Yes	Yes	Yes	Yes	Yes	No	No	High
Aluvaala	2019	26655	Yes	Yes	Yes	Yes	Yes	Unclear	Yes	Yes	Yes	Low
Braun	2020	25305	Yes	Yes	Yes	Yes	Yes	Yes	Yes	Yes	Unclear	Low
Cavallin	2020	25624	Yes	Yes	Yes	Yes	Yes	Yes	Yes	No	Yes	Low
Choi	2019	25899	Yes	Yes	Yes	Yes	Yes	Yes	Yes	Yes	Yes	Low
Conde	2020	26082	Yes	Yes	Yes	Yes	Yes	Yes	Yes	Yes	No	Low
Costa	2020	38052	Yes	Yes	Yes	Yes	Yes	Yes	Yes	Yes	No	Low
Ding	2023	26637	Yes	Yes	Yes	Yes	Yes	Yes	Yes	Yes	Yes	Low
Dumpa	2019	26782	Yes	Yes	Yes	Yes	No	Yes	Yes	Yes	Unclear	High
Eftekharian	2021	38564	Yes	Yes	Yes	Yes	No	Yes	Yes	Yes	No	High
Farah	2018	27107	Yes	Yes	Yes	Yes	Yes	Unclear	Yes	Yes	Unclear	High
Faulks	2023	27143	Yes	Yes	Yes	Yes	No	Yes	Yes	Yes	Yes	Low

Fradkin	2022	38937	Yes	Yes	Yes	Yes	Yes	Yes	Yes	Yes	Unclear	Low
Gebremariam	2022	27579	Yes	Yes	Yes	Yes	No	Yes	Yes	No	Yes	High
Goodman	2019	27831	Yes	Yes	Yes	Yes	Yes	Yes	Yes	No	Unclear	High
Guidozzi	2018	27974	Yes	Yes	No	Yes	No	Yes	Yes	Yes	Yes	High
Hahn	2022	39506	Yes	Yes	Yes	Yes	Yes	Yes	Yes	Yes	Yes	Low
Harrison	2018	28231	Yes	Yes	Yes	Yes	Yes	Yes	Yes	Yes	Yes	Low
Herbozo	2021	39702	Yes	Yes	Yes	Yes	Yes	Yes	Yes	Yes	Yes	Low
Howell	2020	28595	Yes	Yes	Yes	Yes	Yes	Yes	Yes	Yes	Unclear	Low
Hua	2023	28630	Yes	Yes	Yes	Yes	Yes	Yes	Yes	Yes	Yes	Low
Jones	2018	29060	Yes	Yes	Yes	Yes	Yes	Yes	Yes	Yes	Yes	Low
Juliana	2022	29105	Yes	Yes	Yes	Yes	Yes	Yes	Yes	Yes	No	Low
Khasawneh	2020	29460	Yes	Yes	Yes	Yes	Yes	Yes	Yes	Yes	Unclear	Low
Kim	2021	29561	Yes	Yes	Yes	Yes	Yes	Yes	Yes	Yes	Yes	Low
Lanier	2021	29945	Yes	Yes	No	Yes	Yes	Yes	Yes	Yes	Unclear	High
Li	2020	30191	Yes	Yes	Yes	Yes	Yes	Yes	Yes	Yes	Unclear	Low
Li	2019	30204	Yes	Yes	Yes	Yes	Yes	Yes	Yes	Yes	No	Low
Lundeby	2020	30521	Yes	Yes	No	Yes	Yes	Yes	Yes	Yes	Yes	Low
Manandhar	2021	30720	Yes	Yes	Unclear	Yes	No	Yes	Yes	Yes	No	High
Massad	2020	30861	Yes	Yes	Yes	Yes	Yes	Yes	Yes	No	Yes	Low
Mdoe	2022	31015	Yes	Yes	Yes	Yes	Yes	Yes	Yes	Yes	Yes	Low
Moshiro	2019	31435	Yes	Yes	Yes	Yes	Yes	No	Yes	Yes	Yes	Low

**Moura**	2020	41969	Yes	Yes	Yes	Yes	Yes	Yes	Yes	Yes	Yes	Low
**Murphy**	2018	31548	Yes	Yes	Yes	Yes	Yes	Yes	Yes	Yes	Yes	Low
**Pokhrel**	2018	42800	Yes	Yes	Unclear	Yes	Yes	Yes	Yes	Yes	Yes	Low
**Reyesa**	2018	33026	Yes	Yes	Yes	Yes	Yes	Yes	Yes	Yes	Yes	Low
**Sackey**	2019	43314	Yes	Yes	Yes	Yes	Yes	Yes	Yes	No	Yes	Low
**Seeni**	2019	33668	Yes	Yes	Yes	Yes	Yes	Yes	Yes	Yes	Yes	Low
**Seid**	2019	43584	Yes	Yes	Yes	Yes	Yes	Yes	Yes	Yes	Yes	Low
**Serra**	2019	33726	Yes	Yes	No	Yes	Yes	No	Unclear	Yes	Yes	High
**Sunny**	2020	44120	Yes	Yes	Yes	Yes	Yes	Yes	Yes	Yes	Yes	Low
**Tadesse**	2021	34592	Yes	Yes	Yes	Yes	Yes	Yes	Yes	No	Yes	Low
**Tette**	2020	34778	Yes	Yes	Yes	Yes	Yes	Yes	Yes	No	Yes	Low
**Tosif**	2020	34920	Yes	Yes	Yes	Yes	Yes	Yes	Yes	Yes	No	Low
**Tosif**	2018	34921	Yes	Yes	Yes	Yes	Yes	Yes	Yes	Yes	Yes	Low
**Wehrmeister**	2019	35522	Yes	Yes	Unclear	Yes	Yes	Yes	Yes	Yes	Yes	Low
**Xu**	2022	35802	Yes	Yes	Yes	Yes	Yes	Yes	Yes	Yes	Yes	Low
**Zafeiri**	2022	36066	Yes	Yes	Yes	Yes	No	Yes	Yes	Yes	Yes	Low
**Ziem**	2023	36269	Yes	Yes	Unclear	Yes	Yes	Yes	Yes	Yes	Yes	Low

Table S5: Number of neonatal admissions per 1000 live births, by WHO region

WHO region	Admission/1000 LB	95% CI
**African**	160.5	122.2 to 198.7
**Western Pacific**	151.4	141.4 to 161.4
**Eastern Mediterranean**	143.0	124.5 to 161.1
**Regions of America**	122.2	113.5 to 130.8
**Southeast Asian**	121.5	0.8 to 282.6
**European**	96.5	71.8 to 121.3

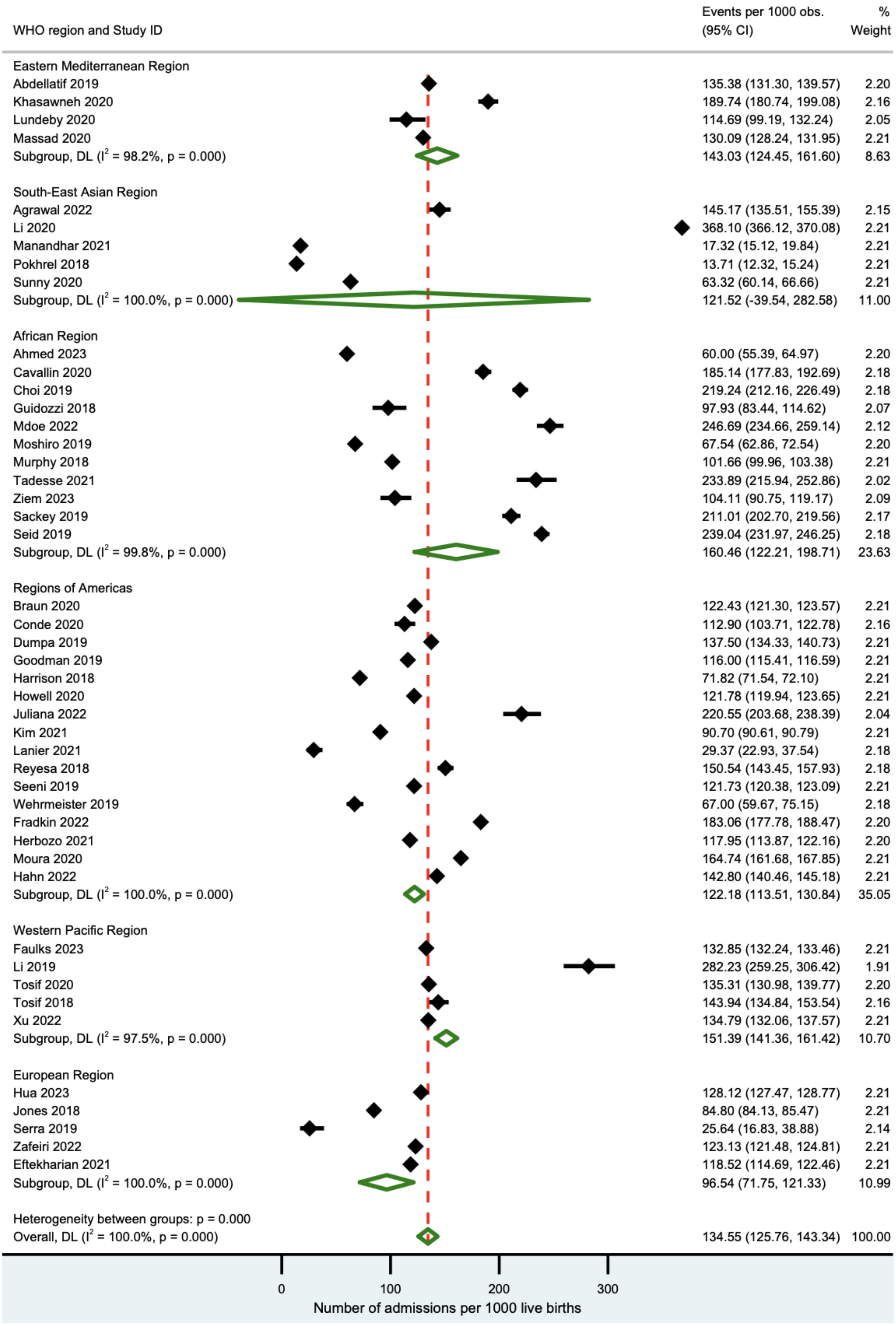

Figure S1: Number of neonatal admissions per 1000 live births, by WHO region

Table S6: Number of neonatal admissions per 1000 live births, by income status

Income status	Admission/1000 LB	95% CI
**HIC**	111.3	101.9 to 120.8
**UMIC**	145.1	129.6 to 160.5
**LMIC**	139.0	62.5 to 215.5
**LIC**	175.3	102.8 to 247.8

HIC: High income countries, UMIC: Upper middle income countries, LMIC: Lower middle income countries, LIC: Lower income countries

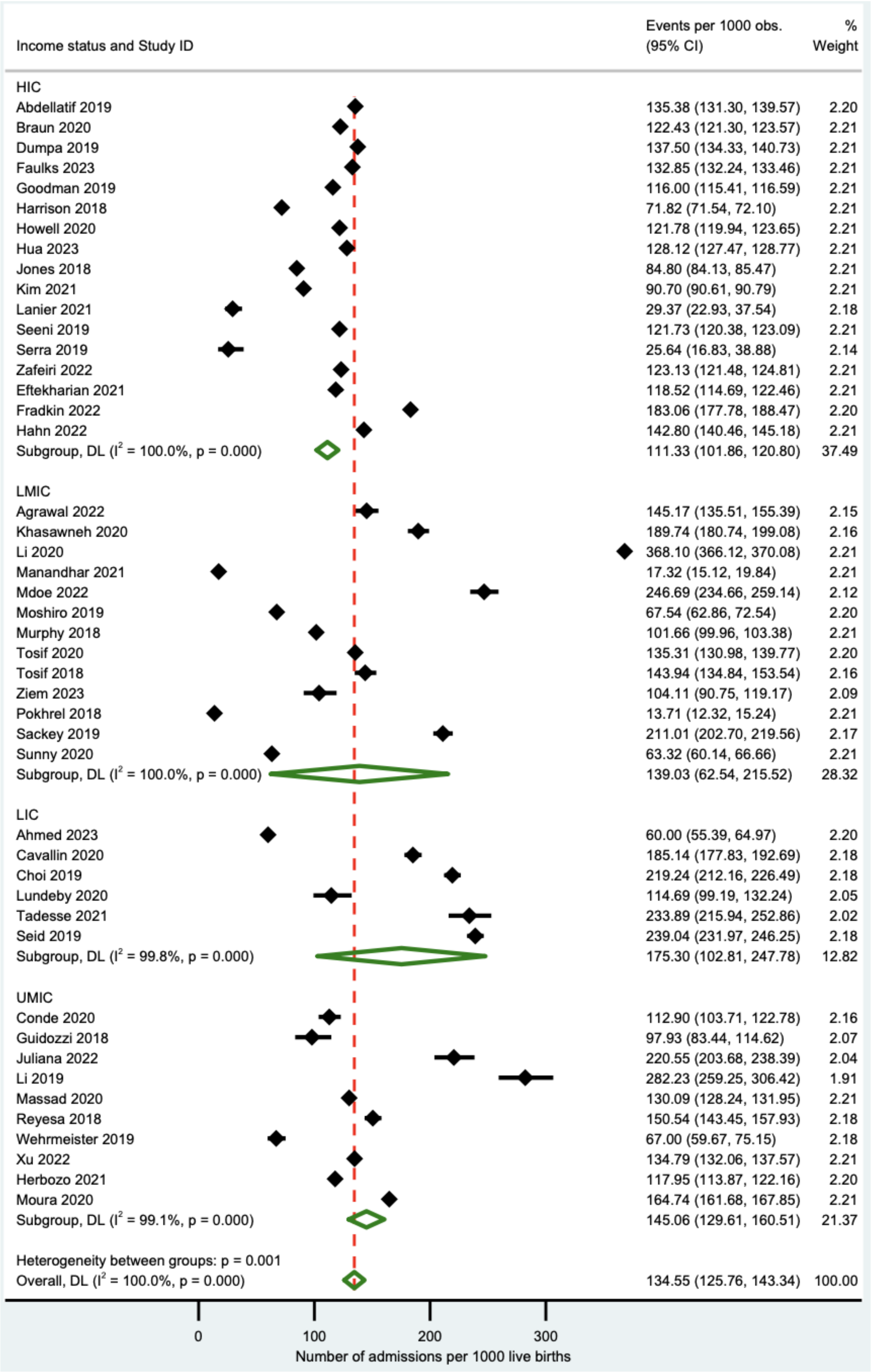

Figure S2: Number of neonatal admissions per 1000 live births, by income status

Table S7: Number of neonatal admissions per 1000 live births, by study setting

Study setting	Admission/1000 LB	95% CI
**Hospital-based**	140.9	111.8 to 170.1
**Population-based**	117.7	106.7 to 128.7

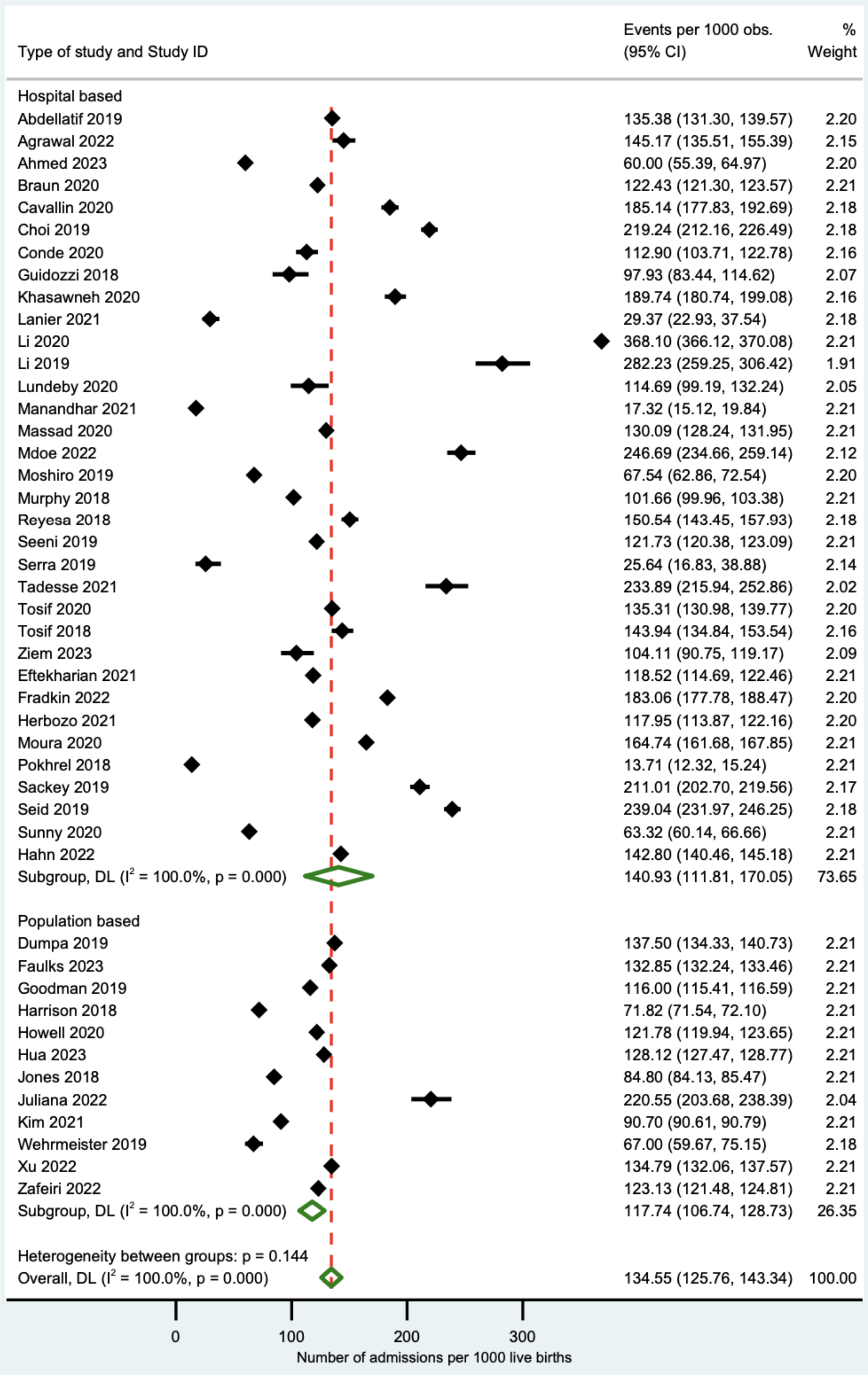

Figure S3: Number of neonatal admissions per 1000 live births, by study setting

Table S8: Number of neonatal admissions per 1000 live births, by level of health facility

Level of facility	Admission/1000 LB	95% CI
**Secondary**	92.1	42.6 to 141.6
**Tertiary**	147.5	115.9 to 179.1

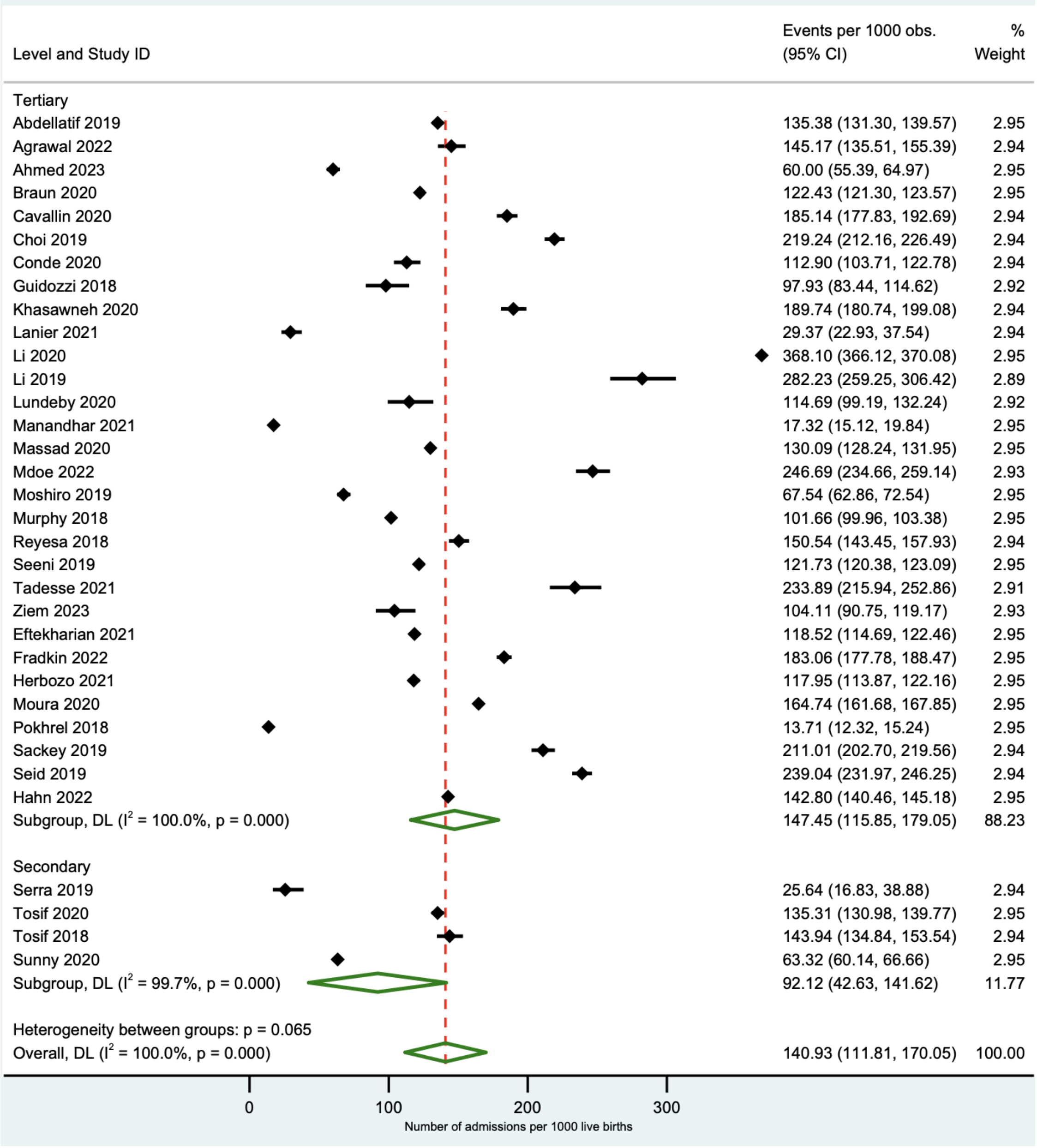

Figure S4: Number of neonatal admissions per 1000 live births, by level of health facility

Table S9: Number of neonatal admissions per 1000 live births, by neonatal mortality rate (NMR) category

NMR per 1000 live births	Admission/1000 LB	95% CI
**≤12**	126.3	118.8 to 133.7
**>12**	149.4	90.5 to 218.2

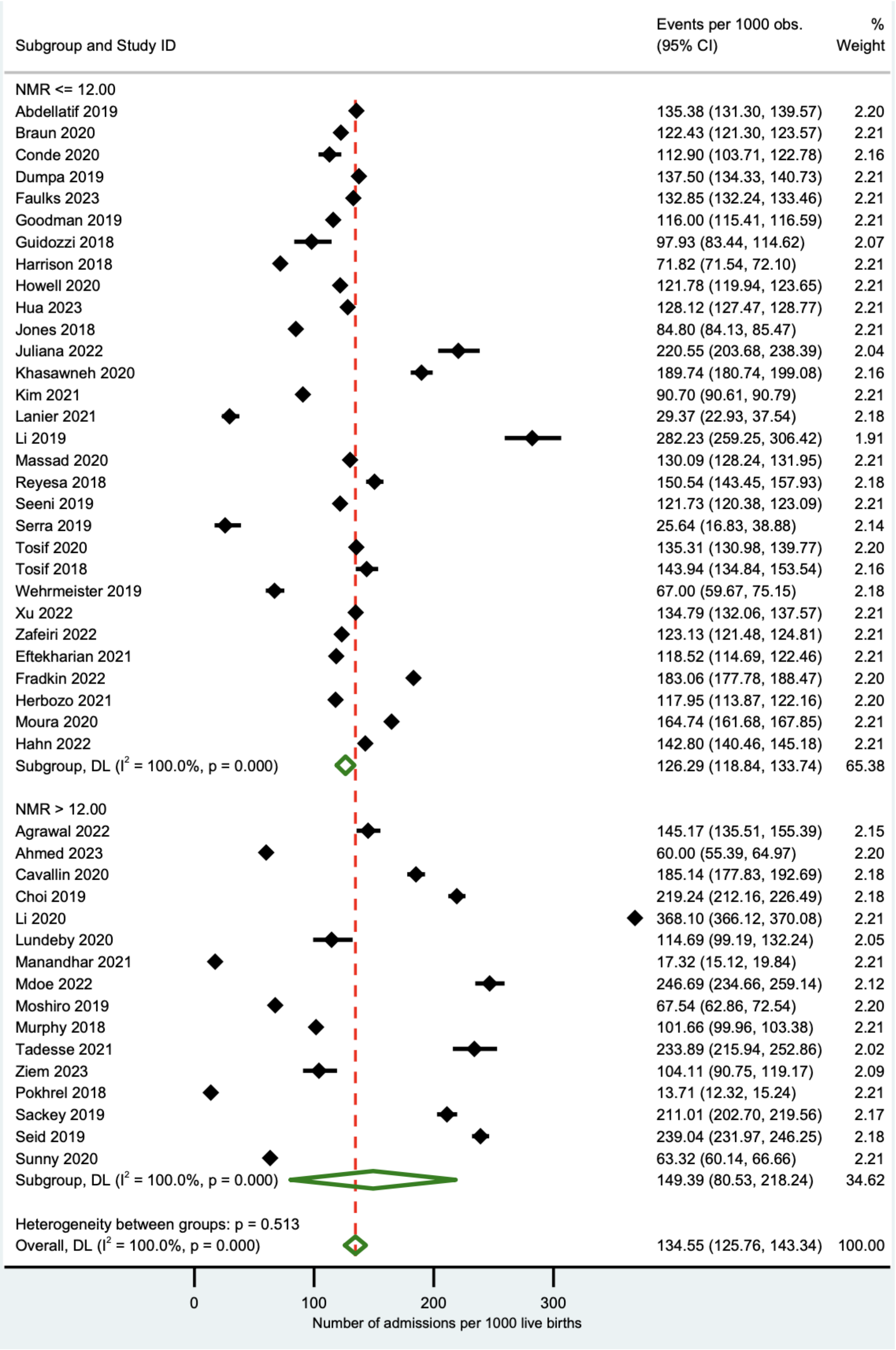

Figure S5: Number of neonatal admissions per 1000 live births, by neonatal mortality rate (NMR) category

Table S10: Number of neonatal admissions per 1000 live births, by risk of bias

Risk of bias	Admission/ 1000 LBs	95% CI
**Low**	147.1	137.2 to 157.0
**High**	75.3	39.7 to 110.9

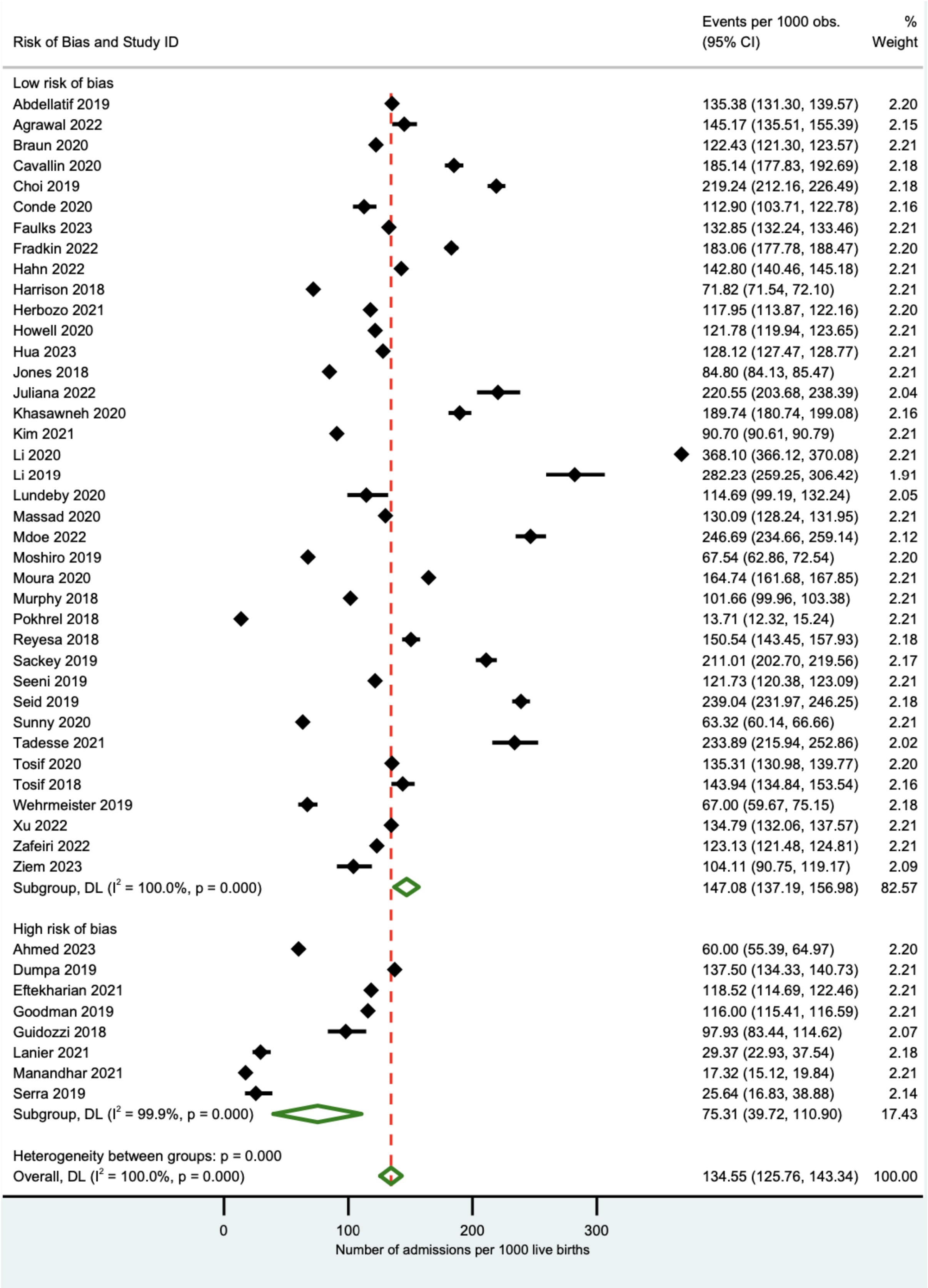

Figure S6: Number of neonatal admissions per 1000 live births, by risk of bias

Table S11: Subgroup analysis of duration of stay by risk of bias

Risk of bias	Duration of stay (days)	95% CI
**Low**	7.06	4.17-9.94
**High**	4.78	3.06-6.50

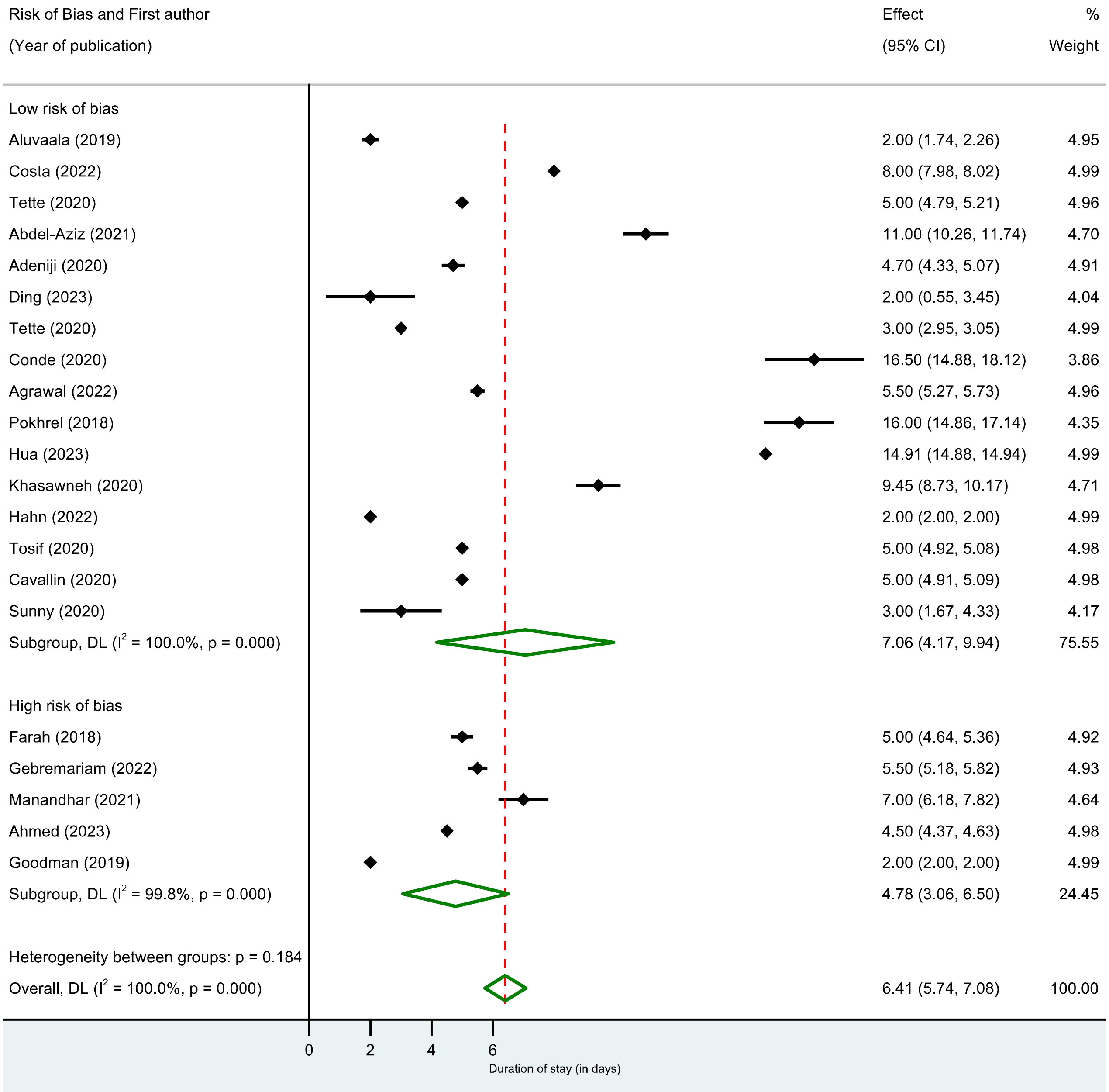

Figure S7: Subgroup analysis of duration of stay by risk of bias

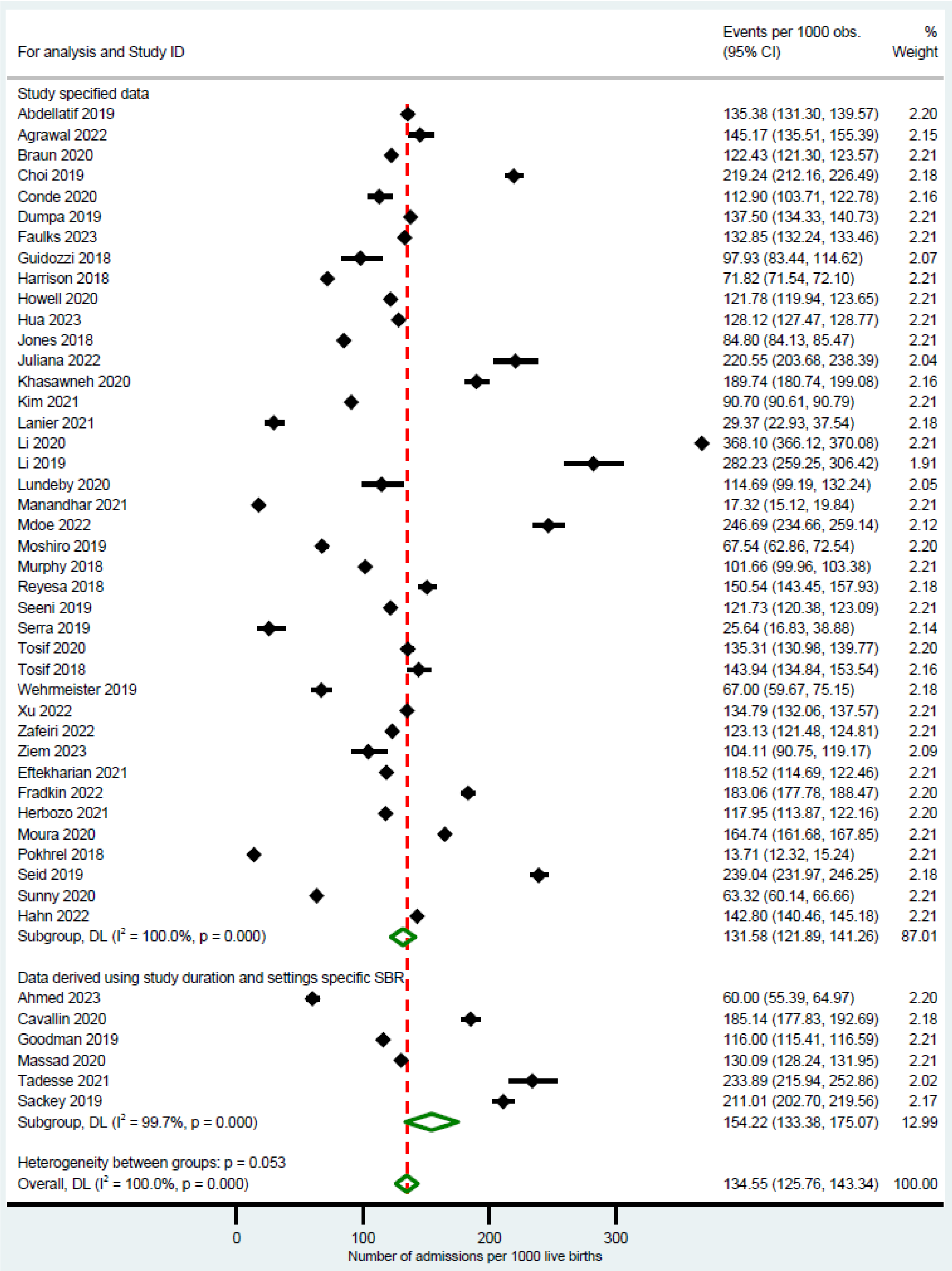

Figure S8: Number of neonatal admissions per 1000 live births, by denominator (study specified or data derived using study duration/settings specific stillbirth rate)

Table S12: GRADE assessment

Question: Number of beds required for small and sick newborns per 1000 live births
Setting: Sick newborn care units and neonatal intensive care units

Certainty assessment							Certainty	Importance
No. of studies	Study design	Risk of bias	Inconsistency	Indirectness	Imprecision	Other considerations		
**Number of admissions of small and sick newborns per 1000 live births**								
46	Non-randomised studies	Not serious[a]	Serious[b]	Not serious[c]	Not serious[d]	Publication bias strongly suspected[e]	⨁◯◯◯ Very low[a,b,c,d,e]	CRITICAL
**Duration of hospital stay**								
20	Non-randomised studies	Serious[g]	Serious[h]	Serious[i]	Not serious[j]	None[k]	⨁◯◯◯ Very low[g,h,i,j,k]	CRITICAL

CI: confidence interval

Explanations

a. Most studies had low risk of bias
b. The number of admissions ranging from 13/1000 LB to 368/1000 LB
c. Few (6/46) showed births which were then converted to live births
d. The confidence interval is narrow 125-143
e. Egger's test p value<0.001
f. Observational study
g. Five out of 20 studies have high risk of bias
h. Duration of stay varied from 2 days to 16 days
i. Few studies mentioned range and mean; which were converted to median and 95% CI
j. The confidence interval is narrow 5.7-7.0 days
k. Egger's test p value 0.07; publication bias not detected

References

1. Abdellatif M, Al-Khabori M, Rahman AU, Khan AA, Al-Farsi A, Ali K. Outcome of Late-onset Neonatal Sepsis at a Tertiary Hospital in Oman. Oman Med J. 2019 Jul;34(4):302–7.

2. Agrawal R, Singh S,Gupta R, Agarwal G, Jain A. Morbidity and Mortality pattern among neonates admitted in a Special Newborn Care Unit of Central India : A Retrospective Observational Study. European Journal of Molecular & Clinical Medicine: Volume 09, Issue 03, 2022, p 476-489.

3. Ahmed AT, Farah AE, Ali HN, Ibrahim MO. Determinants of early neonatal mortality (hospital based retrospective cohort study in Somali region of Ethiopia). Scientific Reports. 2023 Jan 20;13(1):1114.

4. Braun D, Braun E, Chiu V, Burgos AE, Gupta M, Volodarskiy M, et al. Trends in Neonatal Intensive Care Unit Utilization in a Large Integrated Health Care System. JAMA Network Open. 2020 Jun 18;3(6):e205239–e205239.

5. Cavallin F, Bonasia T, Yimer DA, Manenti F, Putoto G, Trevisanuto D. Risk factors for mortality among neonates admitted to a special care unit in a low-resource setting. BMC Pregnancy Childbirth. 2020 Nov 23;20(1):722.

6. Choi J, Urubuto F, Dusabimana R, Agaba F, Teteli R, Kumwami M, et al. Establishing a neonatal database in a tertiary hospital in Rwanda - an observational study. Paediatr Int Child Health. 2019 Nov;39(4):265–74.

7. Conde FUY, Martínez-Villafaña E, Tijerina-Tijerina G, García-Benitez CQ, Oldak-Skvirsky D. Newborn morbidity and mortality in a private hospital in Mexico. Ginecol Obstet Mex. 2020;88(8):525–35.

8. Dumpa V, Avulakunta I, Shelton J, Yu T, Lakshminrusimha S. Induction of labor and early-onset Sepsis guidelines: impact on NICU admissions in Erie County, NY. Maternal Health, Neonatology and Perinatology. 2019 Dec 5;5(1):19.

9. Eftekharian C, Husslein PW, Lehner R. Cesarean Section Rate and Perinatal Outcome Analyses According to Robson’s 10-Group Classification System. Maternal and Child Health Journal. 2021 Sep 1;25(9):1474–81.

10. Faulks MF, McLachlan H, Shafiei T, Forster D, Mogren I, Copnell B, et al. O24 - Perinatal outcomes of socially disadvantaged women in Victoria, Australia: A population-based retrospective cohort study of 1,188,872 singleton births 1999-2016. Women and Birth. 2022 Sep 1;35:11.

11. Fradkin EC, Lafferty MA, Greenspan JS, Aghai ZH. Neonatal intensive care unit admissions before and after the adoption of the baby friendly hospital initiative. The Journal of Maternal-Fetal & Neonatal Medicine. 2022 Feb 16;35(4):657–62.

12. Goodman DC, Ganduglia-Cazaban C, Franzini L, Stukel TA, Wasserman JR, Murphy MA, et al. Neonatal Intensive Care Variation in Medicaid-Insured Newborns: A Population-Based Study. The Journal of Pediatrics. 2019 Jun 1;209:44-51.e2.

13. D F Guidozzi, S Branch, L Chauke. Maternal and fetal outcomes following delivery in a tertiary hospital in Johannesburg, South Africa. S Afr J Obstet Gynaecol 2018;24(3):90-94.

14. Hahn PD, Melvin P, Graham DA, Milliren CE. A Methodology to Create Mother-Baby Dyads Using Data From the Pediatric Health Information System. Hosp Pediatr. 2022 Oct 1;12(10):884–92.

15. Harrison WN, Wasserman JR, Goodman DC. Regional Variation in Neonatal Intensive Care Admissions and the Relationship to Bed Supply. J Pediatr. 2018 Jan;192:73-79.e4.

16. Herbozo C, Julca I, Flores F, Hernandez R, Zegarra J. Incidence and microbiological characteristics of neonatal late onset sepsis in a neonatal intensive care unit in Peru. International Journal of Infectious Diseases. 2021 Jul 1;108:171–5.

17. Howell EM, Johnson P, Cross-Barnet C. Twin Births in Medicaid: Prevalence, Outcomes, Utilization, and Cost in Four States, 2014-2015. Matern Child Health J. 2020 May;24(5):546–51.

18. Hua X, Petrou S, Coathup V, Carson C, Kurinczuk JJ, Quigley MA, et al. Gestational age and hospital admission costs from birth to childhood: a population-based record linkage study in England. Arch Dis Child Fetal Neonatal Ed. 2023 Sep;108(5):485–91.

19. Jones E, Taylor B, Rudge G, MacArthur C, Jyothish D, Simkiss D, et al. Hospitalisation after birth of infants: cross sectional analysis of potentially avoidable admissions across England using hospital episode statistics. BMC Pediatr. 2018 Dec 20;18(1):390.

20. Juliana AE, Holband N, Lissone NPA, Zonneveld R, Evers N, Plötz FB, et al. Incidence of Early and Late Onset Neonatal Sepsis in Suriname: A National Tertiary Hospital Birth-cohort Study. Pediatr Infect Dis J. 2022 Dec 1;41(12):1007–11.

21. Khasawneh W, Sindiani A, Rawabdeh SA, Aleshawi A, Kanaan D. Indications and Clinical Profile of Neonatal Admissions: A Cross-Sectional Descriptive Analysis from a Single Academic Center in Jordan. J Multidiscip Healthc. 2020;13:997–1006.

22. Kim Y, Ganduglia-Cazaban C, Chan W, Lee M, Goodman DC. Trends in neonatal intensive care unit admissions by race/ethnicity in the United States, 2008-2018. Sci Rep. 2021 Dec 10;11(1):23795.

23. Lanier AL, Wiegand SL, Fennig K, Snow EK, Maxwell RA, McKenna D. Neonatal Outcomes After Delivery in Water. Obstet Gynecol. 2021 Oct 1;138(4):622–6.

24. Li JJ, Liu Y, Xie SY, Zhao GD, Dai T, Chen H, et al. Newborn screening for congenital heart disease using echocardiography and follow-up at high altitude in China. Int J Cardiol. 2019 Jan 1;274:106–12.

25. Li G, Bielicki JA, Ahmed ASMNU, Islam MS, Berezin EN, Gallacci CB, et al. Towards understanding global patterns of antimicrobial use and resistance in neonatal sepsis: insights from the NeoAMR network. Arch Dis Child. 2020 Jan;105(1):26–31.

26. Lundeby KM, Heen E, Mosa M, Abdi A, Størdal K. Neonatal morbidity and mortality in Hargeisa, Somaliland: an observational, hospital based study. Pan Afr Med J. 2020;37:3.

27. Manandhar S, Amatya P, Ansari I, Joshi N, Maharjan N, Dongol S, et al. Risk factors for the development of neonatal sepsis in a neonatal intensive care unit of a tertiary care hospital of Nepal. BMC Infect Dis. 2021 Jun 9;21(1):546.

28. Massad S, Tucktuck M, Dar Khawaja R, Dalloul H, Saman KA, Salman R, et al. Improving newborn health in countries exposed to political violence: An assessment of the availability, accessibility, and distribution of neonatal health services at Palestinian hospitals. J Multidiscip Healthc. 2020;13:1551–62.

29. Mdoe P, Katengu S, Guga G, Daudi V, Kiligo IE, Gidabayda J, et al. Perinatal mortality audit in a rural referral hospital in Tanzania to inform future interventions: A descriptive study. PLoS One. 2022;17(3):e0264904.

30. Moshiro R, Perlman JM, Mdoe P, Kidanto H, Kvaløy JT, Ersdal HL. Potential causes of early death among admitted newborns in a rural Tanzanian hospital. PLoS One. 2019;14(10):e0222935.

31. Moura BLA, Alencar GP, Silva ZP da, Almeida MF de. Factors associated with hospitalization and neonatal mortality in a cohort of newborns from the Unified Health System in São Paulo. Rev Bras Epidemiol. 2020;23:e200088.

32. Murphy GAV, Gathara D, Abuya N, Mwachiro J, Ochola S, Ayisi R, et al. What capacity exists to provide essential inpatient care to small and sick newborns in a high mortality urban setting? - A cross-sectional study in Nairobi City County, Kenya. PLoS One. 2018;13(4):e0196585.

33. Pokhrel B, Koirala T, Shah G, Joshi S, Baral P. Bacteriological profile and antibiotic susceptibility of neonatal sepsis in neonatal intensive care unit of a tertiary hospital in Nepal. BMC Pediatr. 2018 Jun 27;18(1):208.

34. Lona Reyes JC, Pérez Ramírez RO, Llamas Ramos L, Gómez Ruiz LM, Benítez Vázquez EA, Rodríguez Patino V. Neonatal mortality and associated factors in newborn infants admitted to a Neonatal Care Unit. Arch Argent Pediatr. 2018 Feb 1;116(1):42–8.

35. Sackey AH, Tagoe LG. Admissions and mortality over a 5-year period in a limited-resource neonatal unit in Ghana. Ghana Med J. 2019 Jun;53(2):117–25.

36. Seeni I, Williams A, Nobles C, Chen Z, Sherman S, Mendola P. Acute air pollution exposure and NICU admission: a case-crossover analysis. Ann Epidemiol. 2019 Sep;37:64-70.e2.

37. Seid SS, Ibro SA, Ahmed AA, Olani Akuma A, Reta EY, Haso TK, et al. Causes and factors associated with neonatal mortality in Neonatal Intensive Care Unit (NICU) of Jimma University Medical Center, Jimma, South West Ethiopia. Pediatric Health Med Ther. 2019;10:39–48.

38. Serra G, Miceli V, Albano S, Corsello G. Perinatal and newborn care in a two years retrospective study in a first level peripheral hospital in Sicily (Italy). Italian Journal of Pediatrics. 2019 Nov 29;45(1):152.

39. Sunny AK, Gurung R, Gurung A, Basnet O, Kc A. Out of Pocket Expenditure for Sick Newborn Care in Referral Hospitals of Nepal. Matern Child Health J. 2020 Feb;24(Suppl 1):57–65.

40. Tadesse M, Hally S, Rent S, Platt PL, Eusterbrock T, Gezahegn W, et al. Effect of a Low-Dose/High-Frequency Training in Introducing a Nurse-Led Neonatal Advanced Life Support Service in a Referral Hospital in Ethiopia. Front Pediatr. 2021;9:777978.

41. Tosif S, Nasi T, Gray A, Sadr-Azodi N, Ogaoga D, Duke T. Assessment of the quality of neonatal care in the Solomon Islands. J Paediatr Child Health. 2018 Feb;54(2):165–71.

42. Tosif S, Jatobatu A, Maepioh A, Subhi R, Francis KL, Duke T. Cause-specific neonatal morbidity and mortality in the Solomon Islands: An assessment of data from four hospitals over a three-year period. J Paediatr Child Health. 2020 Apr;56(4):607–14.

43. Wehrmeister FC, Victora CG, Horta BL, Menezes AMB, Santos IS, Bertoldi AD, et al. Hospital admissions in the first year of life: inequalities over three decades in a southern Brazilian city. Int J Epidemiol. 2019 Apr 1;48(Suppl 1):i63–71.

44. Xu Y, Guo X, Pan Z, Zheng G, Li X, Qi T, et al. Perinatal Risks of Neonatal and Infant Mortalities in a Sub-provincial Region of China: A Livebirth Population-based Cohort Study. BMC Pregnancy Childbirth. 2022 Apr 19;22(1):338.

45. Zafeiri A, Raja EA, Mitchell RT, Hay DC, Bhattacharya S, Fowler PA. Maternal over-the-counter analgesics use during pregnancy and adverse perinatal outcomes: cohort study of 151 141 singleton pregnancies. BMJ Open. 2022 May 3;12(5):e048092.

46. Ziem MS, Saaka FA, Vicar EK, Kuugbee ED, Karikari AB, Ninimiya SY, et al. Pregnancy and the risk of NICU admissions in Nandom Municipality of Ghana: A cross-sectional retrospective study. Health Sci Rep. 2023 Jan;6(1):e1070.

47. Abdel-Aziz SM; H Enas A; Shalaby, Amira M. Study on Inborn and Outborn Neonatal Admissions in Relation to Gestational Maturity in Neonatal Intensive Care Unit at a Tertiary Care University Hospital in Upper Egypt. Journal of Child Science. 2021 Nov 22;11(01):e287–95.

48. Adeniji EO, Kuti BP, E Elusiyan JB. Prevalence, risk factors, and outcome of hospitalization of neonatal hyperglycemia at a Nigerian health facility. Niger J Clin Pract. 2020 Jan;23(1):71–8.

49. Aluvaala J, Collins GS, Maina B, Mutinda C, Wayiego M, Berkley JA, et al. Competing risk survival analysis of time to in-hospital death or discharge in a large urban neonatal unit in Kenya. Wellcome Open Res. 2019;4:96.

50. MFS Costa, C Magluta, SCS Gomer. Perfil dos prestadores de serviço em relação aos procedimentos de cuidado aos recém-nascidos de risco nas bases de dados do Sistema Único de Saúde. Cad. Saúde Pública 2020; 36(4):e00219618.

51. Ding L, Rodean J, Leyenaar JK, Coon ER, Mahant S, Gill PJ, et al. Characterization of Birth Hospitalizations in the United States. Hosp Pediatr. 2023 May 1;13(5):426–39.

52. Elmi Farah A, Abbas AH, Tahir Ahmed A. Trends of admission and predictors of neonatal mortality: A hospital based retrospective cohort study in Somali region of Ethiopia. PLoS One. 2018;13(9):e0203314.

53. Gebremariam H, Tesfai B, Tewelde S, Abay S, Tekeste D, Kibreab F. Demographic, Clinical Profile and Outcomes of Neonates Admitted to Neonatal Intensive Care Unit of Dekemhare Hospital, Eritrea. BMC Pediatr. 2022 Dec 15;22(1):716.

54. Tette EMA, Nartey ET, Nuertey BD, Azusong EA, Akaateba D, Yirifere J, et al. The pattern of neonatal admissions and mortality at a regional and district hospital in the Upper West Region of Ghana; a cross sectional study. PLoS One. 2020;15(5):e0232406.

55. Al-shehri H, Dahmash DT, Rochow N, Alturki B, Alrajhi D, Alayed F, Alhazani F, Alsuhibany H, Naser AY. Hospital Admission Profile of Neonates for Conditions Originating in the Perinatal Period in England and Wales Between 1999–2020: An Ecological Study. Int J Gen Med. 2022;15:1973-1984.

56. Bajaj M, Sharma J, Mahajan S, Sharma M, Sharma PK. To Retrospectively Review and Assess the Survival Rate of Newborns Admitted over 3 years to a Newborn Intensive Care Unit at a Tertiary Care Institute in Northern India. Journal of Clinical Neonatology [Internet]. 2020;9(4). Available from: https://journals.lww.com/jocn/fulltext/2020/09040/to_retrospectively_review_and_assess_the_survival.7.aspx

57. Eshete A, Abiy S. When Do Newborns Die? Timing and Cause-Specific Neonatal Death in Neonatal Intensive Care Unit at Referral Hospital in Gedeo Zone: A Prospective Cohort Study. Int J Pediatr. 2020;2020:8707652.

58. Hadgu FB, Gebretsadik LG, Mihretu HG, Berhe AH. Prevalence and Factors Associated with Neonatal Mortality at Ayder Comprehensive Specialized Hospital, Northern Ethiopia. A Cross-Sectional Study. Pediatric Health Med Ther. 2020;11:29–37.

59. Hanson C, Singh S, Zamboni K, Tyagi M, Chamarty S, Shukla R, et al. Care practices and neonatal survival in 52 neonatal intensive care units in Telangana and Andhra Pradesh, India: A cross-sectional study. PLoS Med. 2019 Jul;16(7):e1002860.

60. Menalu MM, Gebremichael B, Desta KW, Kebede WM, Tarekegn FN, Mulu GB, et al. Time to death and its predictors among neonates who were admitted to the neonatal intensive care unit at tertiary hospital, Addis Ababa, Ethiopia: Retrospective follow up study. Front Pediatr. 2022;10:913583.

61. Mengistu BA, Yismaw AE, Azene ZN, Mihret MS. Incidence and predictors of neonatal mortality among neonates admitted in Amhara regional state referral hospitals, Ethiopia: prospective follow up study. BMC Pediatrics. 2020 Apr 1;20(1):142.

62. Merscher Alves MB, Conté N, Diallo B, Mamadou M, Delamou A, John O, et al. "Assessing Today for a Better Tomorrow": An observational cohort study about quality of care, mortality and morbidity among newborn infants admitted to neonatal intensive care in Guinea. PLoS One. 2021;16(8):e0254938.

63. Meshram RM, James A. Neonatal Outcome of Twins and Singleton Neonates: An Experience from Tertiary Care Teaching Center of Eastern Maharashtra, India. Journal of Clinical Neonatology [Internet]. 2022;11(3). Available from: https://journals.lww.com/jocn/fulltext/2022/11030/neonatal_outcome_of_twins_and_singleton_neonates_.4.aspx

64. Moise IK. Causes of Morbidity and Mortality among Neonates and Children in Post-Conflict Burundi: A Cross-Sectional Retrospective Study. Children (Basel). 2018 Sep 8;5(9).

65. Mokuolu OA, Adesiyun OO, Ibrahim OR, Suberu HD, Ibrahim S, Bello SO, et al. Appraising Neonatal Morbidity and Mortality in a Developing Country Categorized by Gestational Age Grouping and Implications for Targeted Interventions. Front Pediatr. 2022;10:899645.

66. Monoghan R, Crowe S. Timely Presence of Mother with their Newborns on Admission to PICU. Ir Med J. 2023 Jan 19;(No.1):4.

67. Nyishime M, Borg R, Ingabire W, Hedt-Gauthier B, Nahimana E, Gupta N, et al. A retrospective study of neonatal case management and outcomes in rural Rwanda post implementation of a national neonatal care package for sick and small infants. BMC Pediatr. 2018 Nov 12;18(1):353.

68. Ojima WZ, Olawade DB, Awe OO, Amusa AO. Factors Associated with Neonatal Mortality among Newborns Admitted in the Special Care Baby Unit of a Nigerian Hospital. J Trop Pediatr. 2021 Jul 2;67(3):fmab060.

69. Opare-Asamoah K, Koffuor GA, Abdul-Mumin A, Sulemana BM, Saeed M, Quaye L. Clinical Characteristics, Medication Prescription Pattern, and Treatment Outcomes at the Neonatal Intensive Care Unit of a Tertiary Health-Care Facility in Ghana. J Res Pharm Pract. 2021 Mar;10(1):30–7.

70. Orsido TT, Asseffa NA, Berheto TM. Predictors of Neonatal mortality in Neonatal intensive care unit at referral Hospital in Southern Ethiopia: a retrospective cohort study. BMC Pregnancy Childbirth. 2019 Feb 28;19(1):83.

71. Srivastava S, Kushwaha S, Swaroop V. Morbidity Patterns In Neonates Managed In Sick Newborn Care Units Of Lucknow. European Journal of Molecular & Clinical Medicine, 2022; 9(6): 2050-60.

72. Tolossa T, Wakuma B, Mengist B, Fetensa G, Mulisa D, Ayala D, et al. Survival status and predictors of neonatal mortality among neonates admitted to Neonatal Intensive care Unit (NICU) of Wollega University referral hospital (WURH) and Nekemte Specialized hospital, Western Ethiopia: A prospective cohort study. PLoS One. 2022;17(7):e0268744.
